# Knockdown of THOC1 reduces the proliferation of hepatocellular carcinoma and increases the sensitivity to cisplatin

**DOI:** 10.1186/s13046-020-01634-7

**Published:** 2020-07-15

**Authors:** Shijiao Cai, Yunpeng Bai, Huan Wang, Zihan Zhao, Xiujuan Ding, Heng Zhang, Xiaoyun Zhang, Yantao Liu, Yan Jia, Yinan Li, Shuang Chen, Honggang Zhou, Huijuan Liu, Cheng Yang, Tao Sun

**Affiliations:** 1 State Key Laboratory of Medicinal Chemical Biology and College of Pharmacy, Nankai University, Haihe Education Park, 38, Tongyan Road, Tianjin, 300350 China; 2 Tianjin Key Laboratory of Molecular Drug Research, Tianjin International Joint Academy of Biomedicine, Tianjin, China; 3 College of Life Sciences, Nankai University, Tianjin, China

**Keywords:** THOC1, R-loop, Proliferation, Luteolin, Cisplatin, Sensitivity

## Abstract

**Background:**

Hepatocellular carcinoma (HCC) is one of the most common malignant cancers with poor prognosis and high incidence. The clinical data analysis of liver hepatocellular carcinoma samples downloaded from The Cancer Genome Atlas reveals that the THO Complex 1 (THOC1) is remarkable upregulated in HCC and associated with poor prognosis. However, the underlying mechanism remains to be elucidated. We hypothesize that THOC1 can promote the proliferation of HCC. The present study aims to identify THOC1 as the target for HCC treatment and broaden our sights into therapeutic strategy for this disease.

**Methods:**

Quantitative RT**-**PCR, Western blot, immunofluorescence and immunohistochemistry were used to measure gene and protein expression. Colony formation and cell cycle analysis were performed to evaluate the proliferation. The gene set enrichment analysis were performed to identify the function which THOC1 was involved in. The effects of THOC1 on the malignant phenotypes of hepatocellular cells were examined in vitro and in vivo.

**Results:**

The gene set enrichment analysis reveals that THOC1 can promote the proliferation and G2/M cell cycle transition of HCC. Similarly, experimental results demonstrate that THOC1 promotes HCC cell proliferation and cell cycle progression. The knockdown of THOC1 leads to R-loop formation and DNA damage and confers sensitivity to cisplatin. In addition, in vivo data demonstrate that THOC1 can enhance tumorigenesis by increasing tumor cell proliferation. Furthermore, virtual screening predicts that THOC1 as a direct target of luteolin. Luteolin can induce DNA damage and suppress the proliferation of HCC by targeting THOC1. Furthermore, the inhibition of THOC1 activity by luteolin enhances the chemosensitivity of HCC tumor cells to cisplatin.

**Conclusions:**

THOC1 was identified as a predictive biomarker vital for HCC-targeted treatments and improvement of clinical prognosis. Luteolin combined with cisplatin can effectively suppress HCC tumor growth, indicating a potential and effective therapeutic strategy that uses luteolin in combination with conventional cytotoxic agents for HCC treatment.

## Background

Hepatocellular carcinoma (HCC), one of the most common malignant cancers, is currently the leading cause of cancer death worldwide [1]. Hepatitis B virus infection is regarded as the major risk factor that leads to HCC development in China [2]. Although substantial breakthroughs have been made in the diagnostic and therapeutic strategies of HCC, the prognostic rate of patients with HCC remains poor [3, 4]. Currently, the challenges are understanding the molecular mechanism of HCC development and determining the factors that trigger its progression. Thus, the identification of predictive biomarkers is vital for targeted treatments and improved clinical prognosis.

The THO Complex 1 (THOC1) is part of the THO ribonucleoprotein complex that is cotranscriptionally assembled on nascent RNA transcripts, facilitating its interaction with RNA processing and export factors [5–7]. THOC1 deficiency leads to defects in transcription elongation and pre-mRNA export [8, 9] and accumulation of R-loops, resulting in genome instability [10]. Increasing evidence demonstrates that THOC1 expression is elevated in many human cancers, such as prostate [11], colorectal [12] and lung cancers [13], and associated with poor prognosis. However, THOC1 expression and its prognostic significance in HCC remains unknown.

The R-loop is a special chromosome structure that composed of one single-stranded DNA and another strand that contains a DNA:RNA hybrid. The monoclonal antibody S9.6, which recognizes and binds to DNA:RNA hybrids, is used to detect the R-loops [14]. Originally, the R-loop is considered as a rare byproduct of transcription. Now, the R-loop is known to form in the genome of bacteria, yeast, and higher eukaryotes throughout the cell cycle [15–17]. R-loops are a major threat to genome stability [18]. Therefore, increasing number of factors, which include RNA binding and processing factors, helicase, DNA replication, and repair-related factors, are proposed to prevent R-loop formation in cells [19, 20]. A previous study has reported that THOC1–Sin3A interaction can prevent R-loops [21]. The dysfunction in these factors causes R-loop accumulation, leading to replication stress, DNA damage, genome instability, chromatin alterations, or gene silencing, which are phenomena that frequently occur in cancer.

Surgery is regarded as the most effective therapy for patients with HCC [22]. The overall survival of patients with HCC with surgical resection has been prolonged, but the recurrence rate remains at a high level [23]. Therefore, effective nonsurgical treatments are required to improve the survival of patients with HCC. Systemic chemotherapy is a treatment alternative, but this strategy only provides few benefits to patients mainly due to the extreme chemoresistance of HCC to cytotoxic drugs, such as cisplatin [24]. Therefore, the identification of a novel therapeutic strategy, either used alone or in combination with conventional agents, is urgently needed.

## Methods

### Cell culture

The HCC cell lines (Hep3B, HepG2, PLC/PRF/5, and SK-Hep1) were purchased from Guangzhou Cellcook Biotech Co., Ltd. (China). All cell lines were recently authenticated through cellular morphology, and the short tandem repeat analysis was based on the guideline from American type culture collection (ATCC) (Supplemental Data 1). Hep3B and HepG2 cells were cultured using minimum essential media and Dulbecco’s modified Eagle’s medium, respectively, and PLC/PRF/5, and SK-Hep1 cells were cultured using RPMI-1640 medium supplemented with 10% fetal bovine serum and 1% penicillin–streptomycin at 5% CO_2_ and 37 °C.

### Cell viability assay

The cells were cultured in a 96-well plate at a density of 1 × 10^4^ cells/well for 24 h. After 48 h, the cells were added with 10 μL MTT (5 mg/mL), incubated for another 4 h, and added with 100 μL dimethyl sulfoxide (DMSO). The absorbance at 570 nm was measured using a microplate reader (Multiskan™ FC, Thermo Scientific, Waltham, MA, USA).

### Colony formation assay

The cells were plated in a 6-well plate at a density of 400 cells/well. Approximately 2 weeks of culture was needed to form sizeable colonies. The colonies were fixed with 4% paraformaldehyde for 20 min and stained with 0.1% crystal violet solution. Colonies with > 50 cells per colony were counted. The experiments were conducted in triplicate.

### Cell cycle analysis

The HepG2 and the SK-Hep1 cells that were stably transfected with THOC1 or empty vector were seeded in a 6-well plate, whereas the PLC/PRF/5 and the Hep3B cells were transfected with siTHOC1 or siNC. After 48 h of transfection, the cells were fixed in ice-cold 70% ethanol–phosphate buffered saline (PBS) overnight, stained with propidium iodide/RNase A (KeyGen Biotech, Nanjing, China) for 60 min, and sorted using the BD LSR Fortessa (BD Biosciences). The cell cycle distributions were analyzed using the FlowJo 7.6 software. The experiments were performed in triplicate.

### GSEA

Gene set enrichment analysis (GSEA) was performed to identify pathways associated with THOC1 mRNA expression in the TCGA hepatocellular carcinoma dataset [25]. GSEA software was obtained from the Broad Institute (http://www.broad.mit.edu/gsea).

### Quantitative RT-PCR

Total RNA was isolated using the TRIzol reagent and transcribed into cDNA using the *TransScript*® II all-in-one first-strand cDNA synthesis superMix for qPCR kit (TransGen, Beijing, China). Quantitative RT-PCR was performed using the *TransStart*® top green qPCR supermix (TransGen) on the QuantStudio TM 6 Instrument (Life Technologies). The 2^−ΔΔCT^ method was used to calculate the relative gene expression [26]. GAPDH was used as housekeeping gene [27], the sequences of primers used are listed in Supplemental Table S1. The experiment was performed in triplicate.

### Western blotting

The proteins were extracted through the radio-immunoprecipitation assay (RIPA) buffer that contains protease inhibitor cocktail (Sigma, St. Louis, MO, USA) and phenylmethane-sulfonyl fluoride (Sigma). Protein concentration was determined using the Pierce™ BCA Protein Assay Kit (Thermo Scientific). Proteins were separated by 8–12% Tris-acrylamide gels and transferred onto the Immobilon-P Transfer Membranes (Merck KGaA, Darmstadt, Germany). The membranes were incubated with primary antibodies at 4 °C overnight and secondary antibodies at room temperature for 1 h. The primary antibodies used were as follows: anti-Ki67 (1:1000; Affinity, Cincinnati, OH, USA), anti-PCNA (1:1000; Affinity), anti-TUBLIN (1:1000; Affinity), and anti-THOC1 (11,000; Abcam, Cambridge, UK). The proteins were visualized using the ECL reagent (Millipore) and photographed using an electrophoresis gel imaging system (ChemiScope 6000, CLIX, Shanghai, China).

### Immunofluorescence assay

The HCC cells were seeded in 12-well plates, fixed with 4% formaldehyde, permeabilized with PBS containing 0.1% TritonX-100 for 5 min, and blocked with 5% FBS for 1 h. The cells were incubated with primary antibodies γH2AX (1:500; Merck Millipore), S9.6 (1:500; Kerafast, Boston, MA, USA), and THOC1 (1:200; Abcam) at 4 °C overnight and secondary antibodies coupled with Alexa Fluor 647 or 488 (Invitrogen, Waltham, MA, USA) for 1 h at room temperature. The images were captured using a confocal microscope (Nikon, Japan).

### Molecular docking

The crystal structure of THOC1 was downloaded from the PDB database (PDB code, 1WXP). The Sybyl X1.1 software was used to perform molecular docking. The 3-D structure of the traditional Chinese medicine molecule was generated using the LigPrep. Docking score was used to screen small molecules.

### RNA interference and plasmid transfection

All small interference RNA (siRNA) and THOC1-plasmid were transfected using lipofectamine 2000 (Invitrogen, Grand Island, NY, USA) on the basis of the standard protocol. The PLC/PRF/5 and the Hep3B cells transfected with si-negative control (NC) and siTHOC1 were collected after 72 h. The negative control siRNA and THOC1 siRNA sequences were 5′-CCAAACCUACGAGAAUAAUTT-3′ and 5′- UUCUCCGACGUGUCACGUTT-3′, respectively (Shanghai Genephama Co., Ltd., China). The HepG2 and the SK-Hep1 cells were transfected with vector or THOC1 purchased from Sino Biological Inc. (Beijing, China) and collected after 72 h.

### Lentiviral production

The short hairpin RNA (shRNA) targeting control and THOC1 sequences were as follows: shNC: TTCTCCGAACGTGTCACGT, shTHOC1: GATACCAAACCTACGAGAA. The palindromic DNA oligo was annealed to form a double-strand oligo and then ligated to the linearized pLKD-CMV-EGFP-2A-Puromycin-U6-shRNA (OBIO, Shanghai, China) vector to generate the circled pLKD-CMV-shRNA-Puromycin. The specific primers for full-length THOC1 were summarized as follows: THOC1-F: GCTCTAGAATGTCTCCGACGCCG; THOC1-R: CGGGATCCCTAACTATTTGTCTCATTGTCATTA. The amplified fragments were finally ligated into the pLV-EF1a-MCS-IRES-Blasticidin (Biosettia Inc. San Diego, California, USA) expression vector. The PLC/PRF/5 and the HepG2 cells were infected with a lentivirus carrying pLKD-CMV-shRNA-Puromycin or pLV-EF1a-THOC1-Blasticidin plasmids, followed by the separate selection using puromycin or blasticidin to generate polyclonal cell populations.

### Tumor xenograft

Four- to five-week-old female BALB/c nu/nu mice were raised in specific pathogen-free conditions at Tianjin International Joint Academy of Biomedicine. Afterward, the mice were separated randomly into several groups (*n* = 4). For the knockdown assay, the PLC/PRF/5 cells stably transfected with lenti-sh-THOC1 or lenti-sh-NC were injected subcutaneously into the BALB/c nu/nu mice (2 × 10^6^ cells in 100 μL PBS). For the overexpression assay, the HepG2 cells stably transfected with lenti-THOC1 or lenti-Vector were injected subcutaneously into the BALB/c nu/nu mice (2 × 10^6^ cells in 100 μL PBS). For the target validation experiment, the BALB/c nu/nu mice were inoculated subcutaneously with PLC/PRF/5 cells, which are stably transfected with lenti-sh-THOC1 or lenti-sh-NC (2 × 10^6^ cells in 100 μL PBS). When the tumor volume reached approximately 50 mm^3^, the mice were treated by gavage with 50 mg/kg luteolin daily or with saline as control.

For the combination of drug experiment, the PLC/PRF/5 cells were injected subcutaneously into nude mice (2 × 10^6^ cells in 100 μL PBS). The mice in the experiment groups (*n* = 3) were treated with luteolin (50 mg/kg daily, gavage), cisplatin (3 mg/kg, once every 3 days, intraperitoneal injection), or a combination of luteolin (50 mg/kg daily) and cisplatin (3 mg/kg, once every 3 days, intraperitoneal injection) when the tumor volume reached approximately 50 mm^3^. The mice in the control group were treated with the same volume of saline. Body weight and tumor size were measured every 3 days. Tumor volumes were estimated using the formula: *V* = length×width^2^/2 [28]. Finally, all mice were euthanized simultaneously, and the tumors were subjected to immunohistochemistry (IHC) staining. All animal experiments were performed using the approved protocols of the Institutional Animal Care and Use Committee.

### Patient samples

HCC tissues were collected from 26 patients of Tianjin Medical General Hospital and Tumor Hospital of Tianjin within 5 years. The donor was completely informed of the research protocol. Each specimen was obtained upon securing hospital and individual consent. All tissues were harvested under the highest ethical standards.

### Immunohistochemistry

The tumors were embedded in paraffin and cut into serial transverse sections (5 μm). The slides were deparaffinized and dehydrated before IHC was performed. After blocking with 10% normal goat serum for 20 min, the sections were incubated with primary antibodies, including anti-Ki67 (1:100; Affinity), anti-PCNA (1:100; Affinity), anti-S9.6 (1:100; Kerafast), and anti-THOC1 (1:100; Affinity) at 4 °C overnight. Then, the sections were incubated with horseradish peroxidase (HRP)-conjugated secondary antibodies for 1 h at room temperature, followed by staining with 3,3-diaminobenzidine (DAB) solution and counterstaining with hematoxylin. Photographs were captured using an Olympus light microscope (Nikon, Japan). The expression levels of THOC1, RLOOP, PCNA, and Ki67 were evaluated by two independent investigators.

### Statistical analysis

Data are presented as mean ± SD and visualized using GraphPad software (version 7, GraphPad Software, Inc., La Jolla, CA, USA). Statistical analysis was carried out using SPSS version 23.0 (IBM). Student’s *t* test was performed to evaluate statistical significance between two independent groups. One-way ANOVA was utilized to compare multiple groups of data. Survival curve was analyzed using Kaplan–Meier method with logrank (Mantel-Cox test). Correlation between THOC1 and proliferation markers (PCNA and Ki67) in HCC tissues was calculated using Spearman rank correlation test. *P* value < 0.05 was considered statistically significant.

## Results

### Expression level of THOC1 is closely related to the proliferation of HCC

**Fig. 1:**
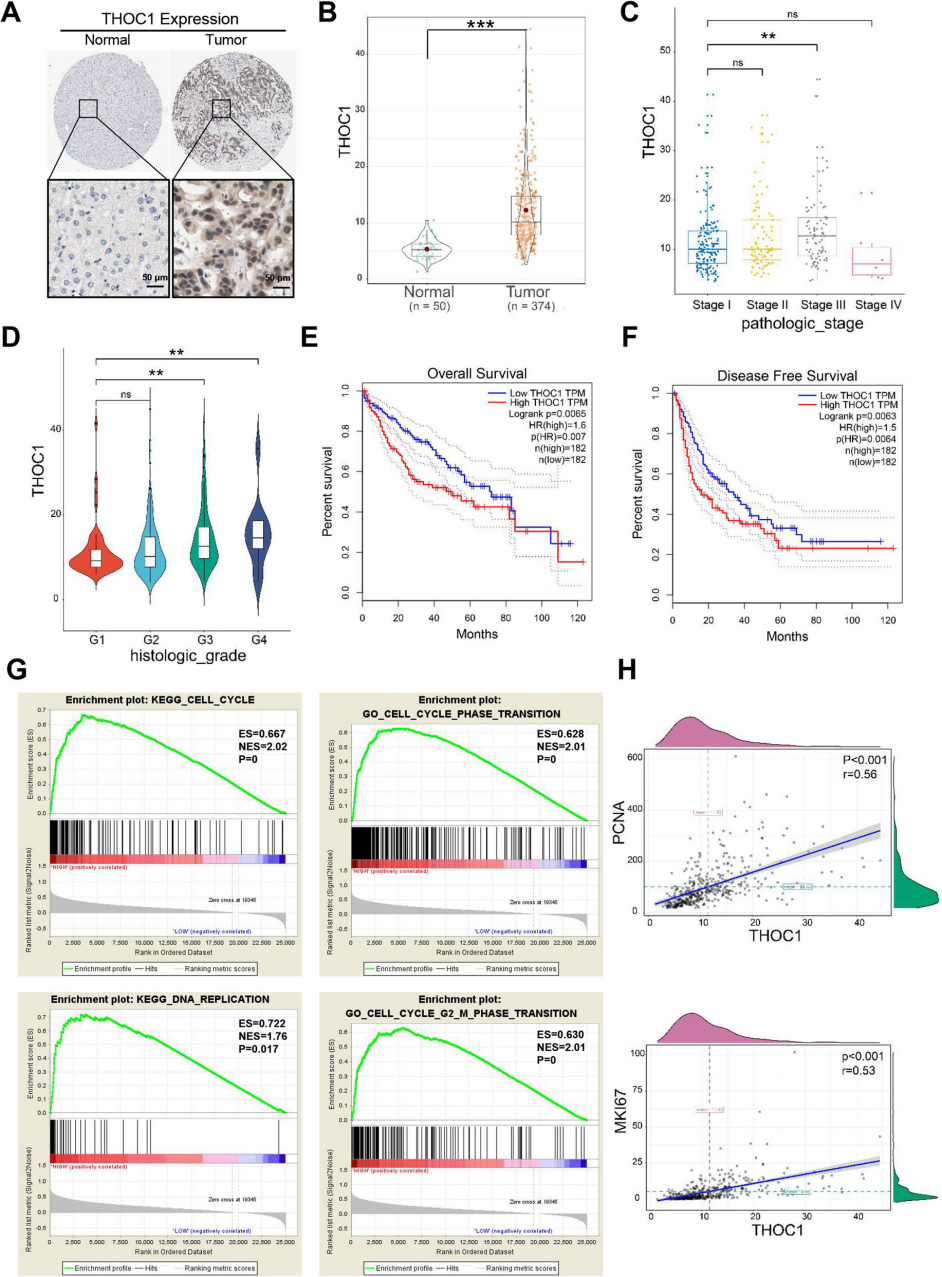
Expression level of THOC1 is closely related to the proliferation of HCC. **a** Representative immunohistochemistry images of THOC1 in normal cells and tumors cited from the Human Protein Atlas. **b** Statistical analysis of THOC1 expression level in normal and tumor cells based on the LIHC samples in The Cancer Genome Atlas (TCGA) database (Student’s *t* test; ***P < 0.001). Statistical analyses of THOC1 expression level in LIHC samples based on (**c**) pathology grade (one-way ANOVA; **P < 0.01) and (**d**) clinical stage (one-way ANOVA; **P < 0.01). **e** Overall survival and (**f**) disease free survival analysis separated by low and high THOC1 expressions in LIHC samples. **g** GSEA based on THOC1 expression. (**h**) Correlation analysis of THOC1 with PCNA and Ki67. **P < 0.01, ***P < 0.001

Clinical data analysis was performed to explore the function of THOC1 in HCC. The representative images of immunohistochemistry downloaded from The Human Protein Atlas database indicated that the THOC1 expression was higher in tumors than that in normal liver tissues (Fig. 1a). Similarly, the clinical data analysis of liver hepatocellular carcinoma (LIHC) samples that were downloaded from The Cancer Genome Atlas (https://portal.gdc.cancer.gov/) showed that the THOC1 expression in tumors (*n* = 374) was significantly higher than that in normal liver tissues (*n* = 50) (Fig. 1b, *P* < 0.001). In addition, THOC1 expression was positively related to pathological grade and clinical stage in LIHC samples (Fig. 1c and d, *P* < 0.05). The overall survival (*P* = 0.0065) and disease free survival (*P* = 0.0063) analyses also indicated that the high expression of THOC1 predicted poor prognosis (Fig. 1e and f). The gene set enrichment analysis (GSEA) indicated that THOC1 can contribute to DNA replication, and G2/M phase transition (Fig. 1g, *P* < 0.05). The correlation analysis of THOC1 and proliferation markers PCNA (*P* < 0.001, *r* = 0.56) and Ki67 (*P* < 0.001, *r* = 0.53) revealed that the expression of THOC1 was positively related to tumor proliferation (Fig. 1h). Furthermore, this result was verified in samples from 26 patients with HCC (Fig. S1). Overall, these data revealed that THOC1 can promote the proliferation of HCC.

### THOC1 promotes HCC cell proliferation and cell cycle progression

**Fig. 2:**
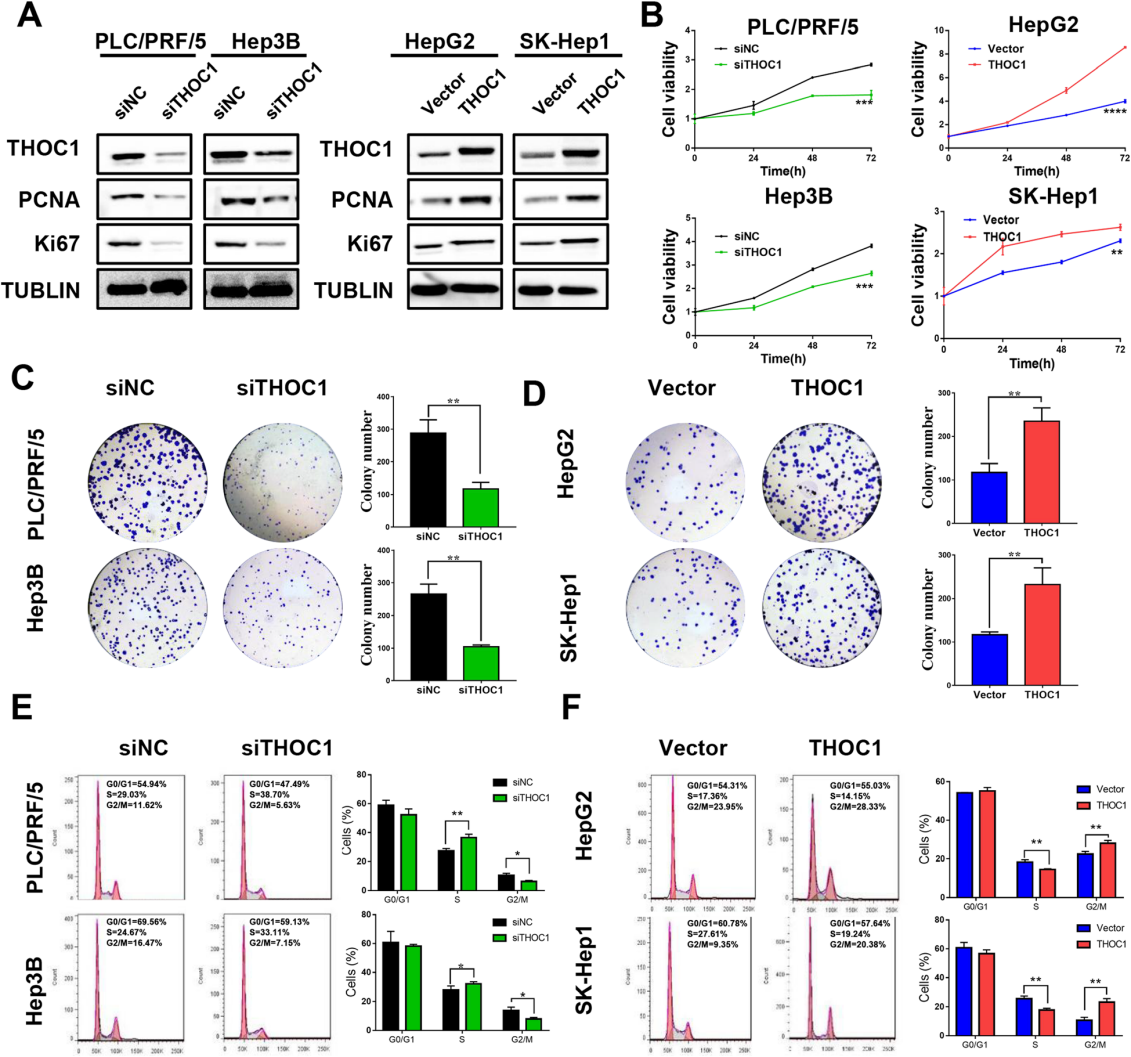
THOC1 promotes HCC cell proliferation and cell cycle progression. **a** Knockdown or ectopic expression efficiency of THOC1 and the expression of proliferation markers were evaluated via Western blot analysis. **b** Cell growth was inhibited by THOC1 knockdown in PLC/PRF/5 and Hep3B cells (left panel), whereas cell viability was dramatically increased by the ectopic expression of THOC1 in HepG2 and SK-Hep1 cells (right panel) (Student’s *t* test; **P < 0.01, ***P < 0.001, ****P < 0.0001). **c** Knockdown of THOC1 inhibited the colony formation in PLC/PRF/5 and Hep3B cells (Student’s *t* test; **P < 0.05). **d** Ectopic expression of THOC1 increased the colony formation in HepG2 and SK-Hep1 cells (Student’s *t* test; **P < 0.05). **e** Cell cycle assay was conducted after THOC1 knockdown in PLC/PRF/5 and Hep3B cells, where the proportion of different cell cycle phases was shown and statistically analyzed (Student’s *t* test; *P < 0.05, **P < 0.01). **f** Cell cycle assay was performed after the ectopic expression of THOC1 in HepG2 and SK-Hep1 cells, where the proportion of cell cycle phases was shown and statistically analyzed (Student’s *t* test; **P < 0.01)

THOC1 was knocked down or overexpressed in HCC cell lines to further verify its effect. Western blot analysis results indicated that THOC1 expression was downregulated in the PLC/PRF/5 and the Hep3B cells transfected with the siTHOC1 plasmid or upregulated in the HepG2 and the SK-Hep1 cells transfected with the THOC1 plasmid (Fig. 2a). Importantly, the expression of crucial proliferation markers (PCNA and Ki67) was reduced during THOC1 knockdown in PLC/PRF/5 and Hep3B cells and increased during the ectopic expression of THOC1 in HepG2 and SK-Hep1 cells (Fig. 2a). Compared with siNC, the knockdown of THOC1 significantly decreased cell viability in PLC/PRF/5 (*P* < 0.001) and Hep3B (*P* < 0.001) cells, whereas the overexpression of THOC1 significantly increased cell viability in HepG2 (*P* < 0.0001) and SK-Hep1 (*P* < 0.01) cells (Fig. 2b). In accordance with these findings, the knockdown of THOC1 reduced the number of colonies in PLC/PRF/5 (*P* < 0.01) and Hep3B (*P* < 0.01) cells compared with siNC (Fig. 2c). By contrast, the ectopic expression of THOC1 enhanced the colony formation ability in HepG2 (*P* < 0.01) and SK-Hep1 (*P* < 0.01) cells (Fig. 2d). The effect of THOC1 in cell cycle progression was further investigated. Results indicated that the knockdown of THOC1 significantly decreased the number of PLC/PRF/5 (6.3% ± 0.71% versus 10.55% ± 1.45%, *P* < 0.05) and Hep3B (7.99% ± 1.03% versus 13.82% ± 2.37%, *P* < 0.05) cells in G2/M phase compared with siNC (Fig. 2e). Conversely, the ectopic expression of THOC1 increased the number of HepG2 (28.04% ± 1.51% versus 22.41% ± 1.40%, *P* < 0.01) and SK-Hep1(23.2% ± 2.53% versus 10.71% ± 2.04%, *P* < 0.01) cells in G2/M phase compared with vector transfection (Fig. 2f). These data confirmed the role of THOC1 in the promotion of proliferation through the regulation of cell cycle progression in HCC cells.

### Knockdown of THOC1 leads to R-loop formation and DNA damage and confers sensitivity to cisplatin

**Fig. 3:**
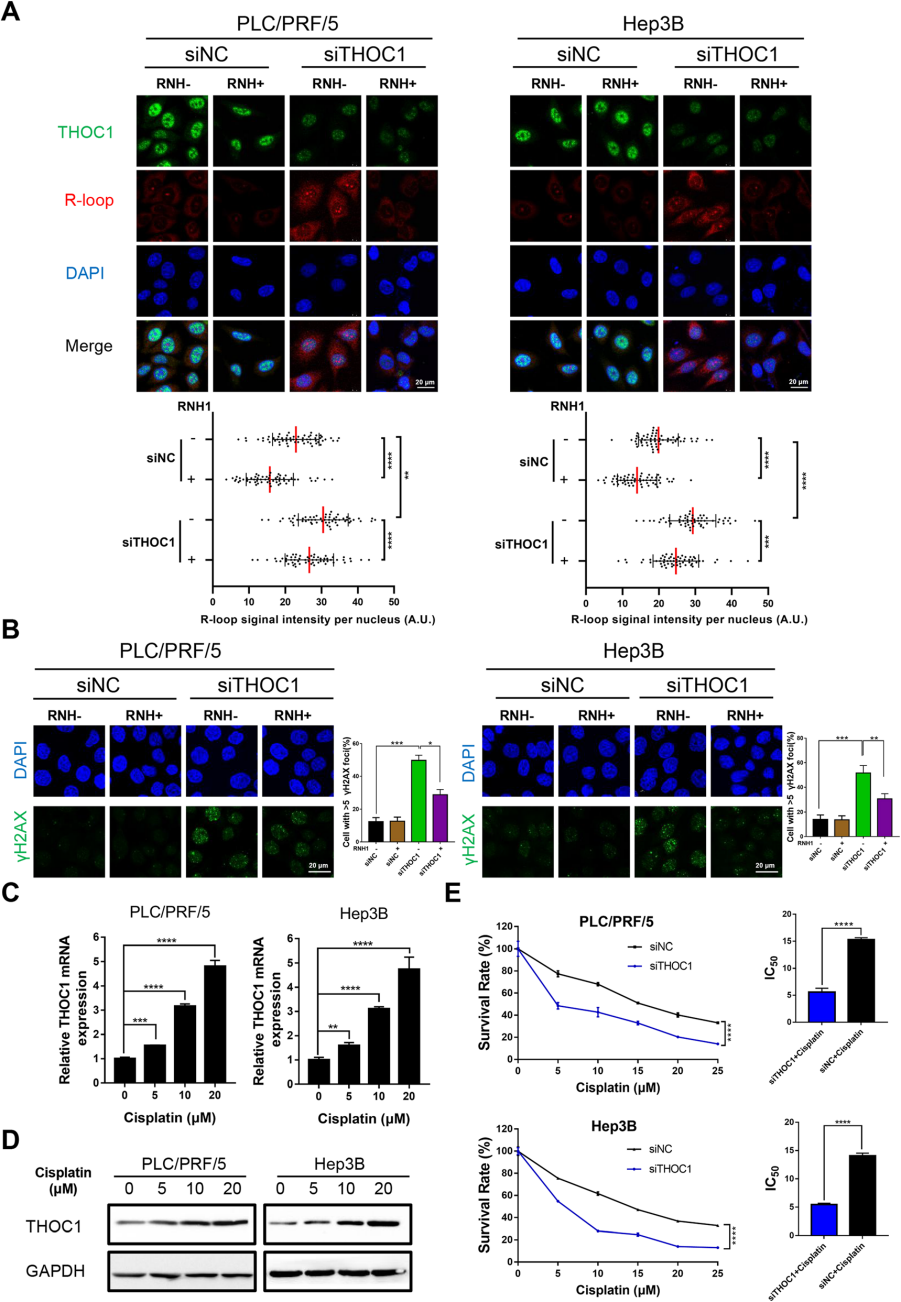
Knockdown of THOC1 leads to increased DNA damage and R-loop formation and confers sensitivity to cisplatin. **a** Immunostaining with R-loop (red) and THOC1 (green) antibodies in PLC/PRF/5 and Hep3B cells transfected with siNC or siTHOC1. The median of the R-loop signal intensity per nucleus after nucleolar signal removal is shown (one-way ANOVA; **P < 0.01, ***P < 0.001, ****P < 0.0001). Scale bar, 20 μm. **b** Immunofluorescence of γH2AX (green) in PLC/PRF/5 and Hep3B cells transfected with siNC or siTHOC1. Percentage of cells that contain > 5 γH2AX foci is shown (one-way ANOVA; *P < 0.05, **P < 0.01, ***P < 0.001). Scale bar, 20 μm. PLC/PRF/5 and Hep3B cells were treated with different concentrations of cisplatin for 48 h, and their expression of THOC1 was detected by (**c**) RT-qPCR (one-way ANOVA; **P < 0.01, ***P < 0.001, ****P < 0.0001) and (**d**) Western blot assay. **e** PLC/PRF/5 and Hep3B cells transfected with siNC or siTHOC1 were treated with different concentrations of cisplatin for 48 h, and their cell viability was evaluated through the MTT assay. The effects of THOC1 knockdown on IC_50_ of cisplatin in PLC/PRF/5 and Hep3B cells were analyzed (Student’s *t* test; ****P < 0.0001)

PLC/PRF/5 and Hep3B cells transfected with siTHOC1 showed a significantly reduced growth rate and cell cycle progression. Then we wondered whether as shown for siTHOC1 cells. The PLC/PRF/5 and the Hep3B cells transfected with siTHOC1 showed a significantly reduced growth rate and cell cycle progression. Then, we wondered whether DNA break accumulation in PLC/PRF/5 and Hep3B cells with THOC1 knockdown were also dependent on R-loops, as shown in siTHOC1 cells [10]. A remarkable accumulation of the S9.6 nuclear signal was detected after THOC1 knockdown in PLC/PRF/5 and Hep3B cells (Fig. 3a, *P* < 0.0001). Importantly, this accumulation was eliminated when RNaseH1 was overexpressed, which normalized the S9.6 signal in THOC1 knockdown cells to that of control cells (Fig. 3a). Furthermore, THOC1 knockdown increased the number of PLC/PRF/5 and Hep3B cells with DNA damage which was indicated by the expression of prominent nuclear foci of γH2AX [29], by 42% (*P* < 0.001) and 48% (*P* < 0.001) compared with siNC transfection, respectively (Fig. 3b). These results indicated that the knockdown of THOC1 resulted in R-loop-induced DNA damage. A previous study reported that in THO-depleted human cells, the formation of R-loops lead to a single-stranded DNA that is susceptible to genotoxic agents [10]. Then, we further investigated whether THOC1 knockdown can sensitize PLC/PRF/5 and Hep3B cells to cisplatin, a chemotherapeutic agent. THOC1 was upregulated in a dose-dependent manner in mRNA and protein levels when PLC/PRF/5 and Hep3B cells were treated with different concentrations of cisplatin (0, 5, 10, and 20 μM) (Fig. 3c and d). We further analyzed the half maximal inhibitory concentrations (IC_50_) of cisplatin in PLC/PRF/5 and Hep3B cells. The IC_50_ of cisplatin decreased in PLC/PRF/5 (5.60 ± 0.70 versus 15.30 ± 0.35, *P* < 0.001) and Hep3B (5.45 ± 0.24 versus14.05 ± 0.49, *P* < 0.001) cells after THOC1 knockdown compared with siNC transfection, indicating that the knockdown of THOC1 can sensitize HCC cells to cisplatin (Fig. 3e). Furthermore, we detected the expression of THOC1 in HepG2/DDP-resistant cell lines. The expression of THOC1 was higher in HepG2/DDP-resistant cell lines than in HepG2 cells (Fig. S2A). We further analyzed the cell viability in HepG2/DDP-resistant cell lines after THOC1 knockdown. Interestingly, CCK-8 assays showed that the knockdown of THOC1 could strengthen DDP-induced cytotoxicity and impaired DDP resistance in HepG2 cell lines (Fig. S2B). These data demonstrated that the knockdown of THOC1 confers sensitivity to cisplatin.

### THOC1 enhances tumorigenesis in vivo

**Fig. 4:**
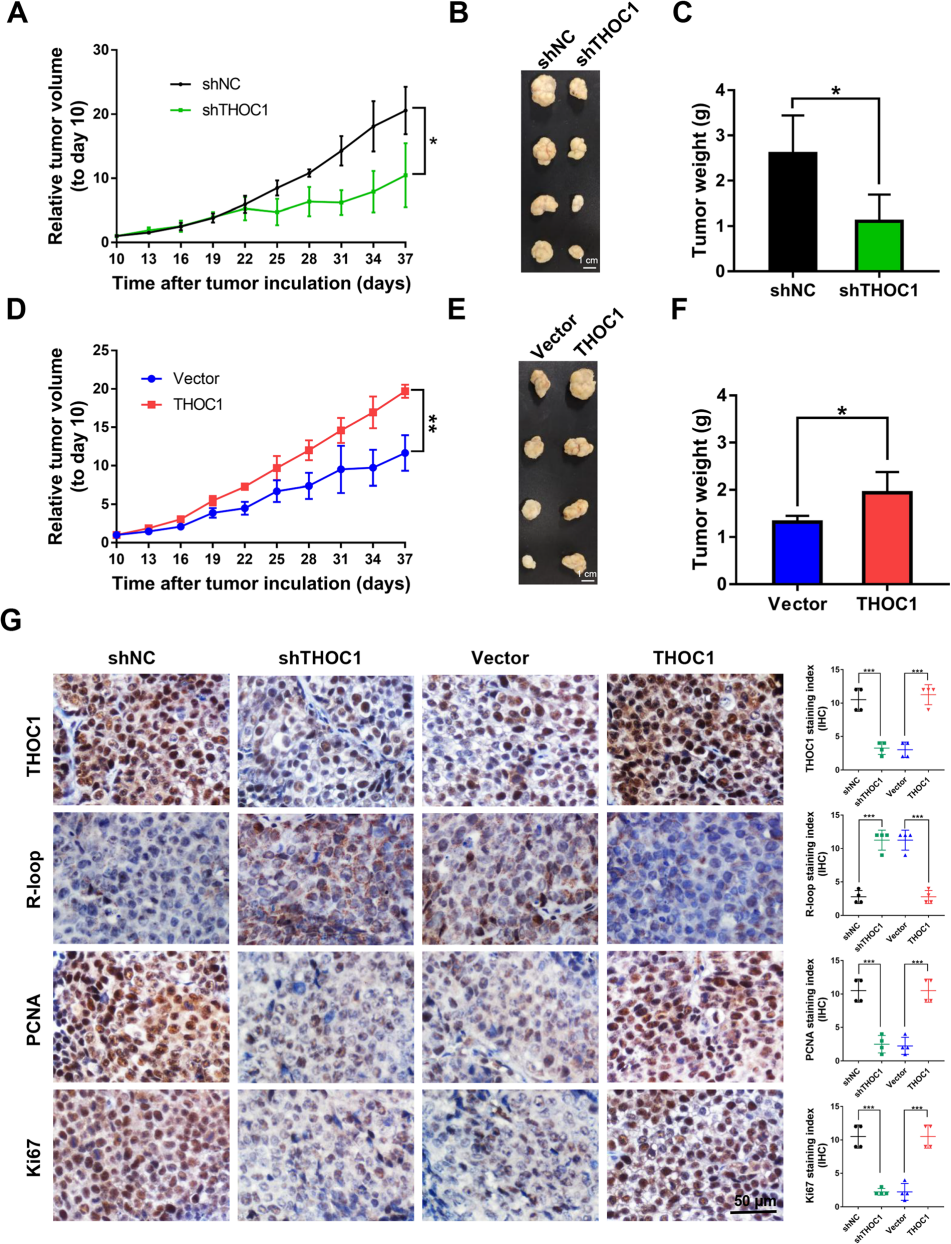
THOC1 enhances tumorigenesis in vivo. **a** Relative tumor volume, (**b**) images of tumor, and (**c**) tumor weight of PLC/PRF/5 stably transfected with shNC or shTHOC1 plasmids in BALB/c nu/nu mice (Student’s *t* test; *P < 0.05). **d** Relative tumor volume, (**e**) images of tumor, and (**f**) tumor weight of THOC1-expressing HepG2 cells in nude mice were compared with those of the control vector-transfected HepG2 cells (Student’s *t* test; *P < 0.05, **P < 0.01). **g** THOC1 protein expression in subcutaneous xenografts was determined by immunohistochemistry. R-loop level was estimated by S9.6 staining, and cell proliferative activity was measured by PCNA and Ki67 staining (Student’s *t* test; ***P < 0.001). Scale bar, 50 μm

THOC1 can promote HCC cell proliferation in vitro. Hence, we further investigated the in vivo tumorigenic ability of THOC1. The tumors derived from PLC/PRF/5 cells with THOC1 knockdown showed growth inhibition compared to their negative control counterparts (Fig. 4a, *P* < 0.05). As a result, the PLC/PRF/5 cells with THOC1 knockdown exhibited reduced tumor size than their control counterparts (Fig. 4b and c, *P* < 0.05). Conversely, the tumors derived from HepG2 cells with THOC1 overexpression showed faster growth compared with their control counterparts (Fig. 4d, *P* < 0.01). Consequently, the HepG2 cells with THOC1 overexpression displayed greater tumor mass than their control counterparts (Fig. 4e and f, *P* < 0.05). The efficiency of THOC1 knockdown and overexpression and was confirmed by IHC staining (Fig. 4g). In line with the in vitro findings, the IHC staining indicated higher R-loop level and lower PCNA and Ki67 levels in PLC/PRF/5 tumors with THOC1 knockdown than those in their control counterparts (Fig. 4g, *P* < 0.001). By contrast, lower R-loop level and higher PCNA and Ki67 levels were observed in HepG2 tumors that overexpressed THOC1 than those in their control counterparts (Fig. 4g, *P* < 0.001). Overall, these data demonstrated that THOC1 can enhance tumorigenesis in vivo by increasing tumor cell proliferation.

### Luteolin reduces HCC proliferation by targeting THOC1 in vitro

**Fig. 5:**
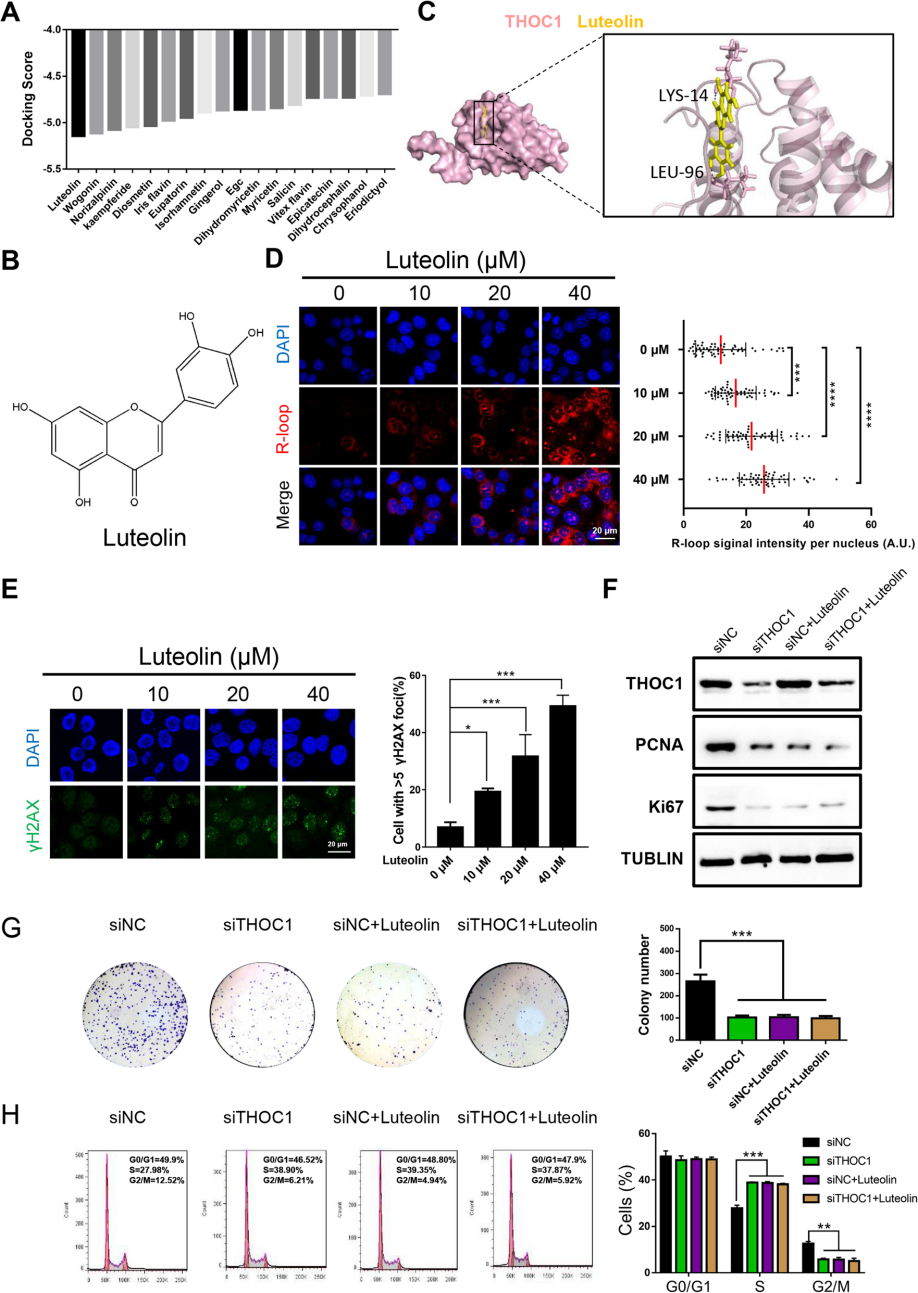
Luteolin reduces HCC proliferation by targeting THOC1 in vitro*.* **a** Molecular docking results of THOC1 with multiple traditional Chinese medicine. **b** Molecular structure of luteolin. **c** Image of molecular dynamics simulation visualizes the combination of luteolin with THOC1. **d** Immunostaining with R-loop (red) in PLC/PRF/5 cells treated with different concentrations of luteolin (0, 10, 20, and 40 μM) for 48 h. Median of the R-loop signal intensity per nucleus after nucleolar signal removal is shown (one-way ANOVA; ***P < 0.001, ****P < 0.0001). Scale bar, 20 μm. **e** Immunofluorescence of γH2AX (green) in PLC/PRF/5 cells treated with different concentrations of luteolin (0, 10, 20, and 40 μM) for 48 h. Percentage of cells that contain > 5 γH2AX foci is shown (one-way ANOVA; *P < 0.05, ***P < 0.001). Scale bar, 20 μm. **f** THOC1 and the proliferation marker (PCNA and Ki67) expressions in PLC/PRF/5 cells transfected with siNC and siTHOC1 plasmids, followed by separate treatment with DMSO or luteolin (20 μM) for 48 h. **g** Colony formation and (**h**) cell cycle assays in PLC/PRF/5 cells transfected with siNC and siTHOC1, followed by separate treatment with DMSO or luteolin (20 μM) for 48 h. The numbers of colonies and proportion of cell cycle phases were compared (one-way ANOVA; **P < 0.01, ***P < 0.001)

Based on the structure of THOC1, lead compounds with high docking scores to THOC1 were selected from the traditional Chinese medicine database by virtual screening (Fig. 5a and b). The molecular dynamics simulation displayed the dynamic binding of luteolin to THOC1 (Fig. 5c). A remarkable and dose-dependent accumulation of the S9.6 nuclear signal was detected in PLC/PRF/5 cells after treatment with different concentrations of luteolin (0, 10, 20, and 40 μM) for 48 h (Fig. 5d, *P* < 0.001). Consequently, the number of PLC/PRF/5 cells with DNA damage was increased by 15% (*P* < 0.05) in the 10 μM luteolin group, by 35% (*P* < 0.001) in the 20 μM luteolin group, and by 47% (*P* < 0.001) in the 40 μM luteolin group compared with that in the 0 μM luteolin group (Fig. 5e). Western blot assay, colony formation, and cell cycle analysis were performed to further explore the effect of luteolin on proliferation. Results clearly showed that THOC1 expression was downregulated in PLC/PRF/5 cells after being transfected with the siTHOC1 compared with siNC (Fig. 5f). Whether THOC1 was knocked down or luteolin (20 μM) alone was added for 48 h, both cases can evidently suppress the expression of proliferation markers (PCNA and Ki67) and inhibit colony formation. Interestingly, when THOC1 was knocked down, the luteolin showed no significant inhibitory effect on the expression of proliferation markers (PCNA and Ki67) and colony formation compared with DMSO (Fig. 5f and g, *P* < 0.001). Moreover, the number of PLC/PRF/5 cells in G2/M phase decreased after the addition of luteolin alone (5.81% ± 0.45% versus 12.57% ± 0.97%, *P* < 0.01) or knockdown of THOC1 (5.73% ± 0.87% versus 12.57% ± 0.97%, *P* < 0.01) compared with that in the control. Nevertheless, no remarkable difference in the siTHOC1 group was observed, regardless whether luteolin treatment was conducted (Fig. 5h). These results demonstrated that luteolin suppressed proliferation in HCC by targeting THOC1.

### Luteolin reduces HCC proliferation by targeting THOC1 in vivo and enhances the antitumor effect of cisplatin

**Fig. 6:**
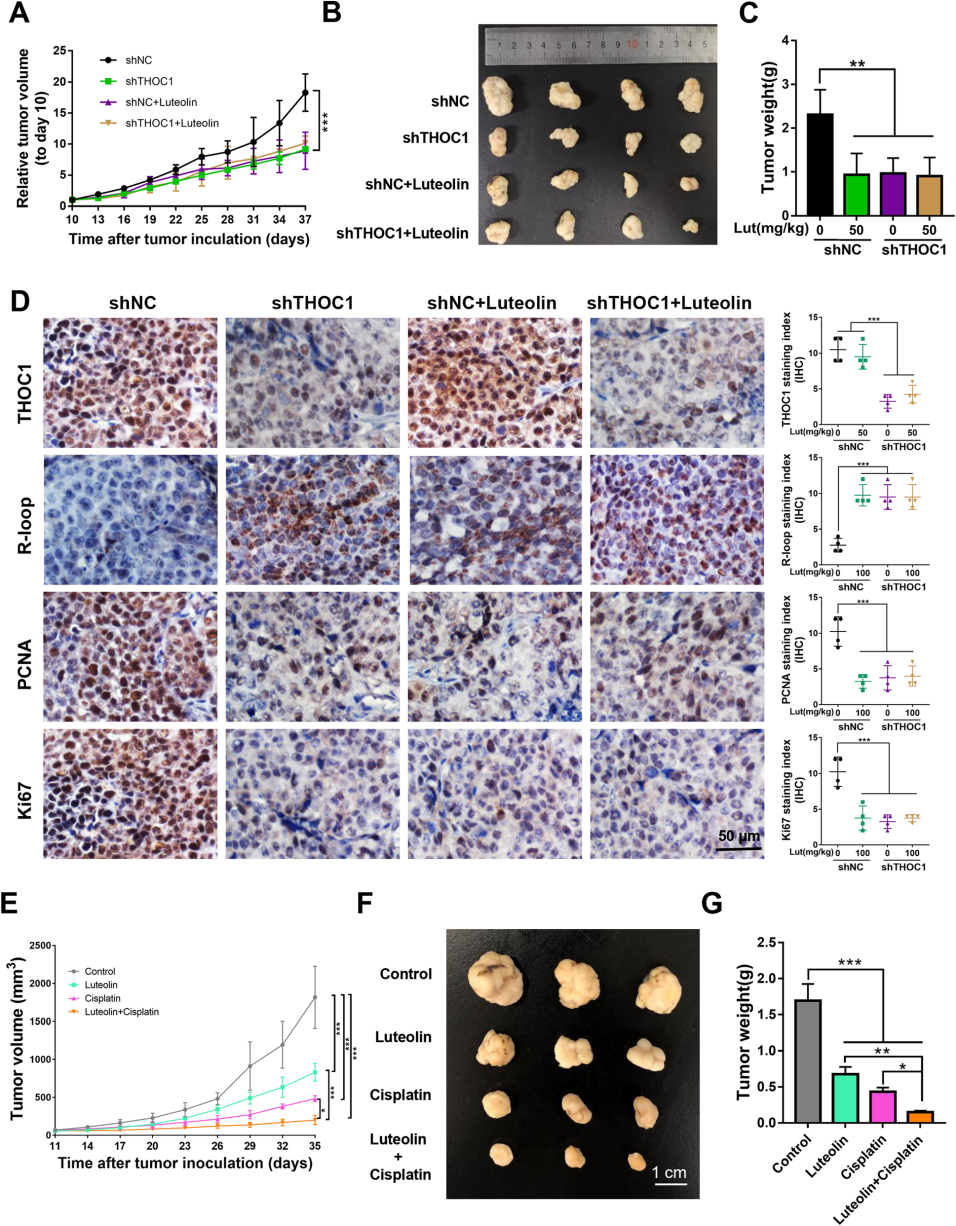
Luteolin reduces HCC proliferation by targeting THOC1 in vivo and enhances the anti-tumor effect of cisplatin. **a** Tumor growth curve, (**b**) representative images of tumor, and (**c**) tumor weight of PLC/PRF/5 cells stably transfected with shTHOC1 or shNC in BALB/c nu/nu mice treated with 50 mg/kg luteolin or saline as control, respectively (one-way ANOVA; **P < 0.01, ***P < 0.001). **d** immunohistochemistry staining indicates the expressions of THOC1, R-loop, and proliferation markers (PCNA and Ki67) in tumors (one-way ANOVA; ***P < 0.001). **e** Tumor growth curve, (**f**) representative images of tumor, and (**g**) tumor weight of PLC/PRF/5-bearing BALB/c nu/nu mice. Luteolin or cisplatin treatment significantly suppressed tumor growth. Furthermore, luteolin can enhance the antitumor effect of cisplatin (one-way ANOVA; *P < 0.05, **P < 0.01, ***P < 0.001)

The BALB/c nu/nu mice subcutaneously injected with PLC/PRF/5 cells, which were stably transfected with lenti-sh-NC or lenti-sh-THOC1 were treated with luteolin or saline as control to estimate the antitumor effect of luteolin. The relative tumor volume, tumor images, and tumor weight demonstrated that the growth of tumor was inhibited in the shTHOC1 group or when 50 mg/kg luteolin alone was administered compared with that in the shNC group. However, the mice in the shTHOC1 group exhibited a slight response to luteolin compared with saline (Figs. 6a–c, *P* < 0.01). These results demonstrated that the antitumor effect of luteolin was exerted in a THOC1-dependent manner. The IHC analysis of PCNA and Ki67 also indicated that the proliferative capacity of tumor was inhibited after luteolin treatment or THOC1 knockdown. Nevertheless, no obvious difference in the proliferative capacity of tumor was observed in the shTHOC1 group regardless of luteolin addition (Fig. 6d, *P* < 0.001). These results indicated that luteolin reduced proliferation by targeting THOC1 in vivo. Furthermore, the antitumor effects of luteolin and cisplatin were assessed in BALB/c nu/nu mice that were subcutaneously injected with PLC/PRF/5 cells. Tumor volume, tumor images, and tumor weight indicated that luteolin and cisplatin can inhibit tumor growth. Moreover, the combination of luteolin and cisplatin enhanced the antitumor effect of cisplatin on tumor size compared with cisplatin alone, indicating the cooperative inhibitory effect of luteolin and cisplatin on tumor growth (Figs. 6e-g, *P* < 0.05).

## Discussion

Our present study elucidated that THOC1 can enhance tumorigenesis in vitro and in vivo*.* Luteolin can suppress the proliferation of HCC by targeting THOC1. Furthermore, the inhibition of THOC1 activity by luteolin has enhanced the chemosensitivity of HCC tumor cells to cisplatin.

THOC1 expression is elevated in breast cancer than in normal epithelia, and its expression level is positively correlated with tumor size and metastasis [30]. In addition, THOC1 is required for tumor cells that undergo neoplastic transformation [11, 31]. Similarly, THOC1 was upregulated in patients with HCC, suggesting the critical role of the oncogenic function of THOC1 during HCC development. Our study indicated that high THOC1 expression was significantly associated with poor prognosis. Therefore, THOC1 can be regarded as prognostic biomarker that can be used to provide better risk assessment of prognosis. GSEA indicated that THOC1 promoted HCC cell proliferation through the promotion of the G2/M phase transition. The role of THOC1 in HCC in vitro and in vivo was investigated. The knockdown of THOC1 in PLC/PRF/5 and Hep3B cells significantly inhibited cell growth. Conversely, the ectopic expression of THOC1 in HepG2 and SK-Hep1 cells significantly increased cell proliferation ability compared with the empty vector.

The knockdown of THOC1 led to an increase in R-loop level, which was in line with the results of a previous study [21]. Moreover, previous research has reported that R-loops result in the generation of DNA double-strand breaks (DSBs), which in turn leads to hypermutation, cell cycle arrest, or even cell death [32]. Currently, the role of THOC1 in genome instability had not been reported in HCC. The present study first reported that THOC1 promoted the proliferation of HCC through the prevention of R-loops that cause genome instability. Our study clearly showed an increase in DNA damage, as determined by the increasing γH2AX foci after THOC1 knockdown.

A previous study indicated that the viability of normal cells is largely unaffected by THOC1 loss. Normal cells that lack Thoc1 cannot be transformed by forced expression of E1A and Ha-ras, suggesting that Thoc1 may be important for neoplastic transformation. Cancer cells require higher levels of THOC1 for survival than normal cells [31]. Thus, we hypothesized that in clinical practice, patients will experience few long-term side effects after THOC1 alteration. THOC1 may represent a novel and effective molecular target for cancer therapy. In our study, the knockdown of THOC1 activity is expected to inhibite the viability of cancer cells. Given that the mechanism of THOC1is novel, the utilization of THOC1 as a therapeutic target may provide discernible clinical responses and potential opportunities for novel combination therapy. For example, yeast cells that are deficient in the THOC1 orthologue HPR1 are highly sensitive to DNA damage [33]. Human cancer cell lines with THOC1 depletion show increased sensitivity to camptothecin and cisplatin [34]. These observations suggest that the inhibition of THOC1 in human cancer cells will increase sensitivity to genotoxic therapy.

R-loop formation sensitizes cells to chemotherapeutics that induce DSBs [35]. In THO-depleted human cells, R-loops are formed and leads to a single-stranded DNA, which is more susceptible to spontaneous damage by genotoxic agents or activation-induced cytidine deaminase [10]. In line with this finding, the present study found that the knockdown of THOC1 can sensitize HCC cells to cisplatin through the analysis of IC_50_ of cisplatin in PLC/PRF/5 and Hep3B cells. Furthermore, the combination of luteolin and cisplatin exhibited a synergy effect in suppressing tumor growth in vivo*.*

## Conclusions

Overall, THOC1 was identified as a predictive biomarker vital for HCC-targeted treatments and improvement of clinical prognosis. Luteolin combined with cisplatin can effectively suppress HCC tumor growth, indicating a potential and effective therapeutic strategy that uses luteolin in combination with conventional cytotoxic agents for HCC treatment.

## Supplementary information

**Additional file 1: Figure S1.** Expression of THOC1 is positively correlated with the expression of proliferation markers (PCNA and Ki67). A. Representative IHC staining images for PCNA and Ki67 in THOC1-negative and -positive HCC tissues (scale bar = 50 μm). B. PCNA and THOC1 staining was quantified, and the correlation was analyzed (correlation coefficient: *r* = 0.6046, *P* < 0.01). C. Ki67 and THOC1 staining was quantified, and the correlation was analyzed (correlation coefficient: r = 0.5526, *P* < 0.01). **Figure S2.** Effects of cisplatin in HepG2/DDP-resistant cell lines after THOC1 knockdown. A. Western blot analysis was performed to analyze the expression levels of THOC1 in HepG2 and HepG2/DDP-resistant cell lines. B. Cell viability in the HepG2/DDP-resistant cell line after THOC1 knockdown was assessed via CCK-8 assays. **** *P* < 0.0001.

**Additional file 2: Table S1.** Primers used for RT-PCR.

**Additional file 3.**

## Data Availability

All data generated or analyzed during this study are included either in this article or in the supplementary information files.

## Abbreviations

ATCC: American type culture collection
DAB: 3, 3-diaminobenzidine
DMSO: Dimethyl sulfoxide
DSBs: DNA double-strand breaks
GSEA: Gene Set Enrichment Analysis
HCC: Hepatocellular carcinoma
HRP: Horseradish peroxidase
IC50: Half-maximal inhibitory concentrations
IHC: Immunohistochemistry
LIHC: Liver hepatocellular carcinoma
NC: Negative control
shRNA: short hairpin RN
siRNA: small interference RNA
TCGA: The Cancer Genome Atlas
THOC1: THO Complex 1
