# Correction: Knockdown of THOC1 reduces the proliferation of hepatocellular carcinoma and increases the sensitivity to cisplatin

**DOI:** 10.1186/s13046-024-03208-3

**Published:** 2024-10-14

**Authors:** Shijiao Cai, Yunpeng Bai, Huan Wang, Zihan Zhao, Xiujuan Ding, Heng Zhang, Xiaoyun Zhang, Yantao Liu, Yan Jia, Yinan Li, Shuang Chen, Honggang Zhou, Huijuan Liu, Cheng Yang, Tao Sun

**Affiliations:** 1 State Key Laboratory of Medicinal Chemical Biology and College of Pharmacy, Nankai University, Haihe Education Park, 38, Tongyan Road, Tianjin, 300350 China; 2 Tianjin Key Laboratory of Molecular Drug Research, Tianjin International Joint Academy of Biomedicine, Tianjin, China; 3 College of Life Sciences, Nankai University, Tianjin, China

**Correction: J Exp Clin Cancer Res 39, 135 (2020)**

10.1186/s13046-020-01634-7

Following the publication of the original article [1], the author identified errors in the images of Figs. 4G and 6D which were unintentionally caused during the figure assembly process. Specifically:

- Figure 4G: R-loop-Vector.
- Figure 6D: R-loop-shNC and Ki67-shNC.

The corrected figures are provided below:

The corrections do not affect the overall results, discussion, or conclusion of the article.

**Incorrect Fig. 4**

**Fig. 4:**
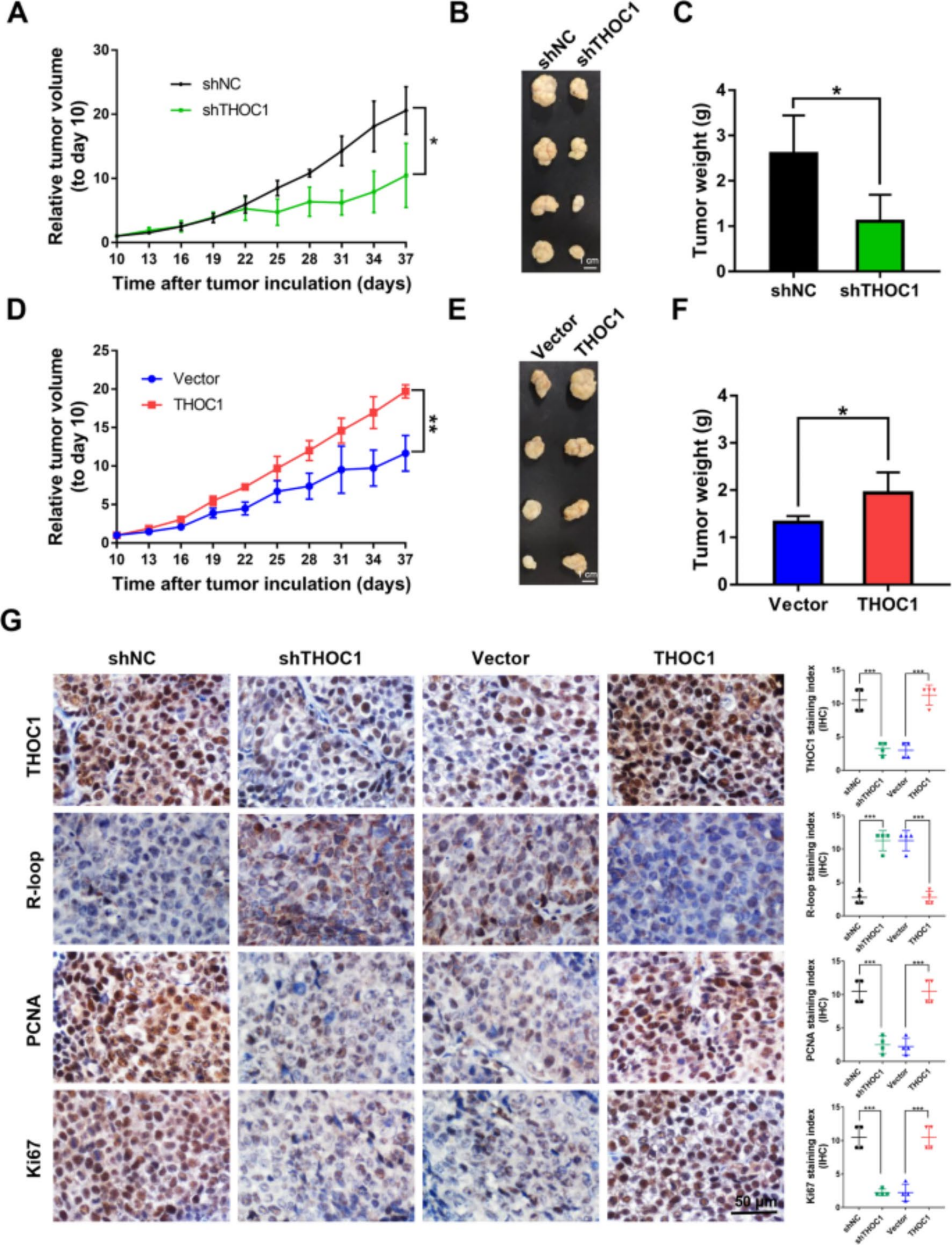
THOC1 enhances tumorigenesis in vivo. **a** Relative tumor volume, (**b**) images of tumor, and (**c**) tumor weight of PLC/PRF/5 stably transfected with shNC or shTHOC1 plasmids in BALB/c nu/nu mice (Student’s *t* test; *P < 0.05). **d** Relative tumor volume, (**e**) images of tumor, and (**f**) tumor weight of THOC1-expressing HepG2 cells in nude mice were compared with those of the control vector-transfected HepG2 cells (Student’s *t* test; *P < 0.05, **P < 0.01). **g** THOC1 protein expression in subcutaneous xenografts was determined by immunohistochemistry. R-loop level was estimated by S9.6 staining, and cell proliferative activity was measured by PCNA and Ki67 staining (Student’s *t* test; ***P < 0.001). Scale bar, 50 μm

**Correct Fig. 4**

**Fig. 4:**
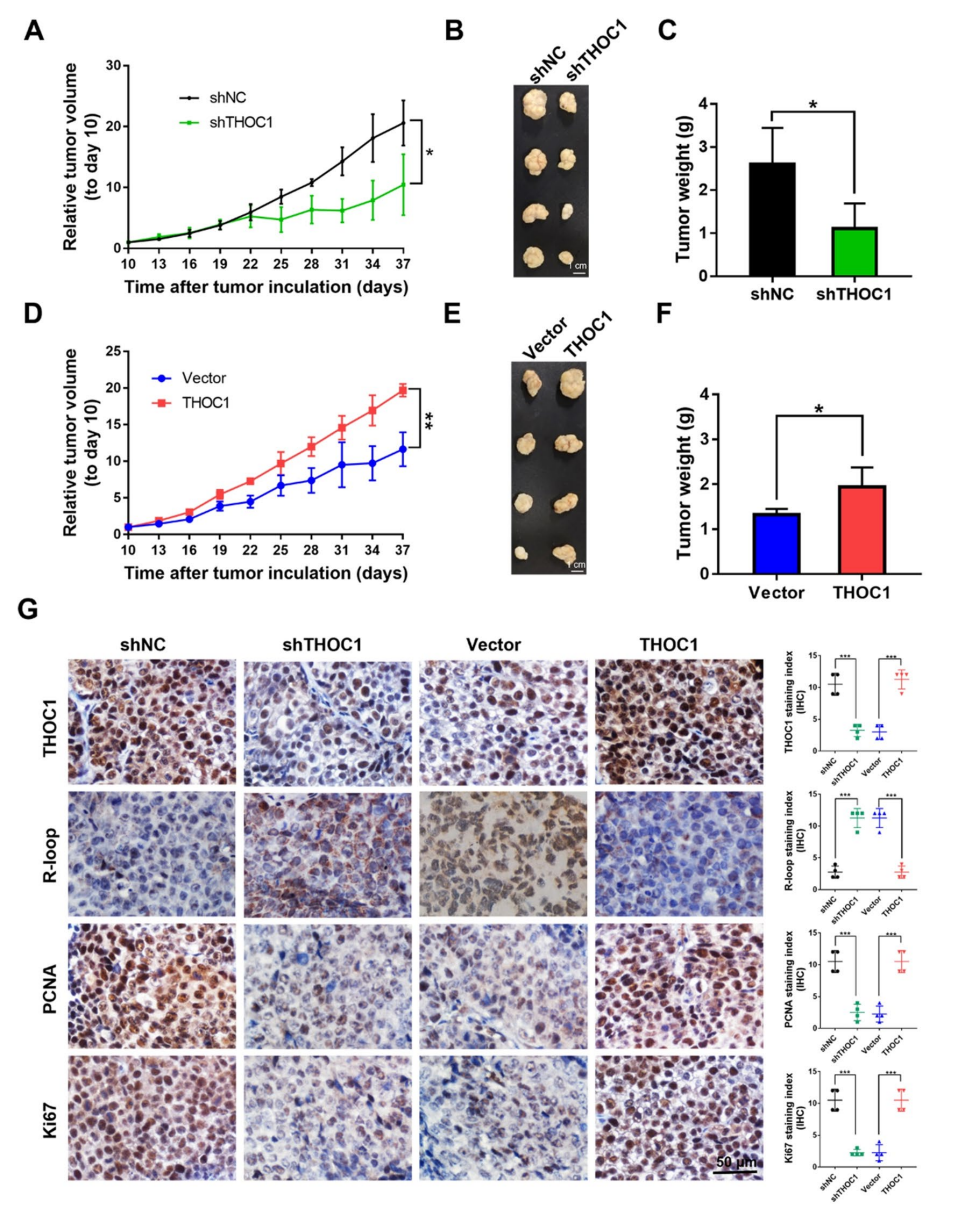
THOC1 enhances tumorigenesis in vivo. **a** Relative tumor volume, (**b**) images of tumor, and (**c**) tumor weight of PLC/PRF/5 stably transfected with shNC or shTHOC1 plasmids in BALB/c nu/nu mice (Student’s *t* test; *P < 0.05). **d** Relative tumor volume, (**e**) images of tumor, and (**f**) tumor weight of THOC1-expressing HepG2 cells in nude mice were compared with those of the control vector-transfected HepG2 cells (Student’s *t* test; *P < 0.05, **P < 0.01). **g** THOC1 protein expression in subcutaneous xenografts was determined by immunohistochemistry. R-loop level was estimated by S9.6 staining, and cell proliferative activity was measured by PCNA and Ki67 staining (Student’s *t* test; ***P < 0.001). Scale bar, 50 μm

**Incorrect Fig. 6**

**Fig. 6:**
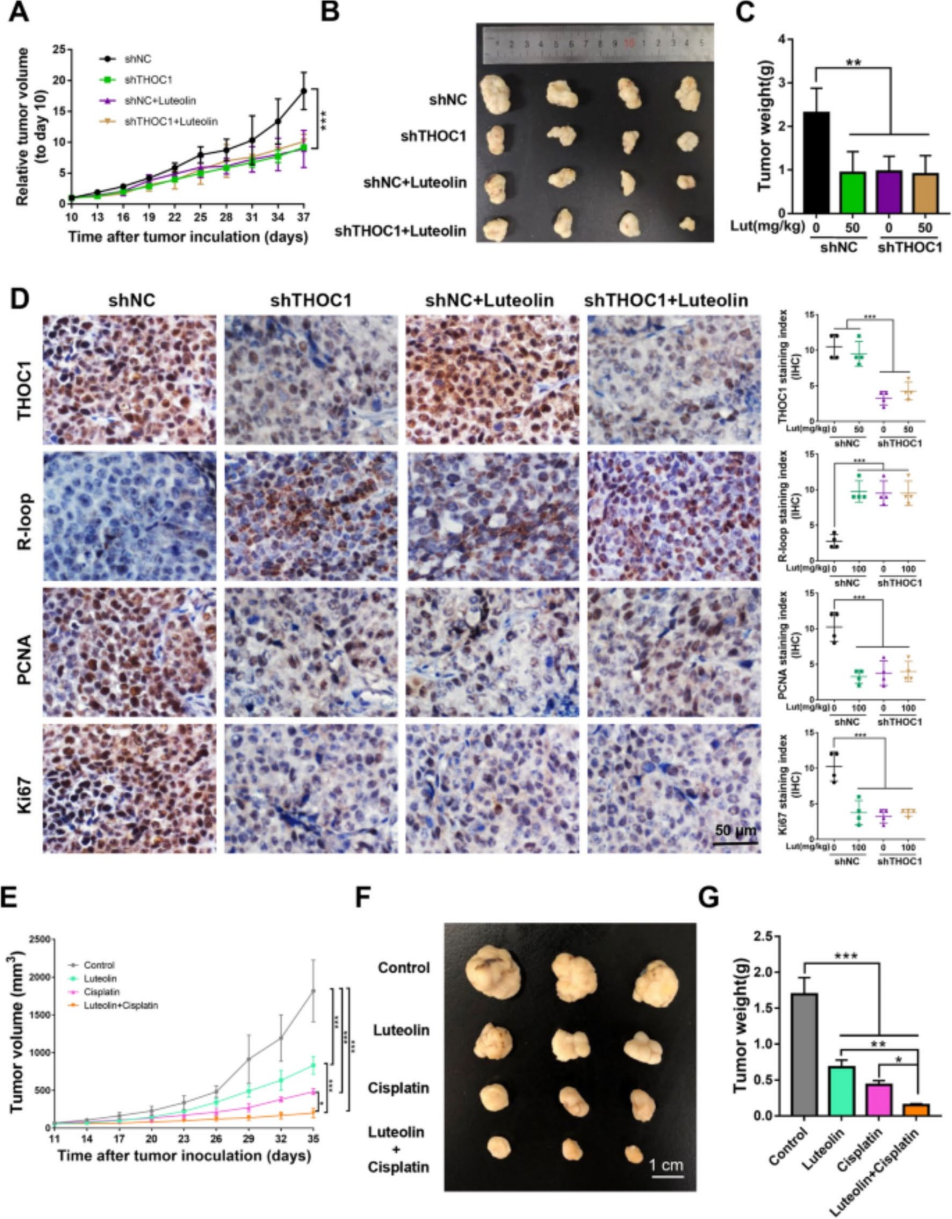
Luteolin reduces HCC proliferation by targeting THOC1 in vivo and enhances the anti-tumor effect of cisplatin. **a** Tumor growth curve, (**b**) representative images of tumor, and (**c**) tumor weight of PLC/PRF/5 cells stably transfected with shTHOC1 or shNC in BALB/c nu/nu mice treated with 50 mg/kg luteolin or saline as control, respectively (one-way ANOVA; **P < 0.01, ***P < 0.001). **d** immunohistochemistry staining indicates the expressions of THOC1, R-loop, and proliferation markers (PCNA and Ki67) in tumors (one-way ANOVA; ***P < 0.001). **e** Tumor growth curve, (**f**) representative images of tumor, and (**g**) tumor weight of PLC/PRF/5-bearing BALB/c nu/nu mice. Luteolin or cisplatin treatment significantly suppressed tumor growth. Furthermore, luteolin can enhance the antitumor effect of cisplatin (one-way ANOVA; *P < 0.05, **P < 0.01, ***P < 0.001)

**Correct Fig. 6**

**Fig. 6:**
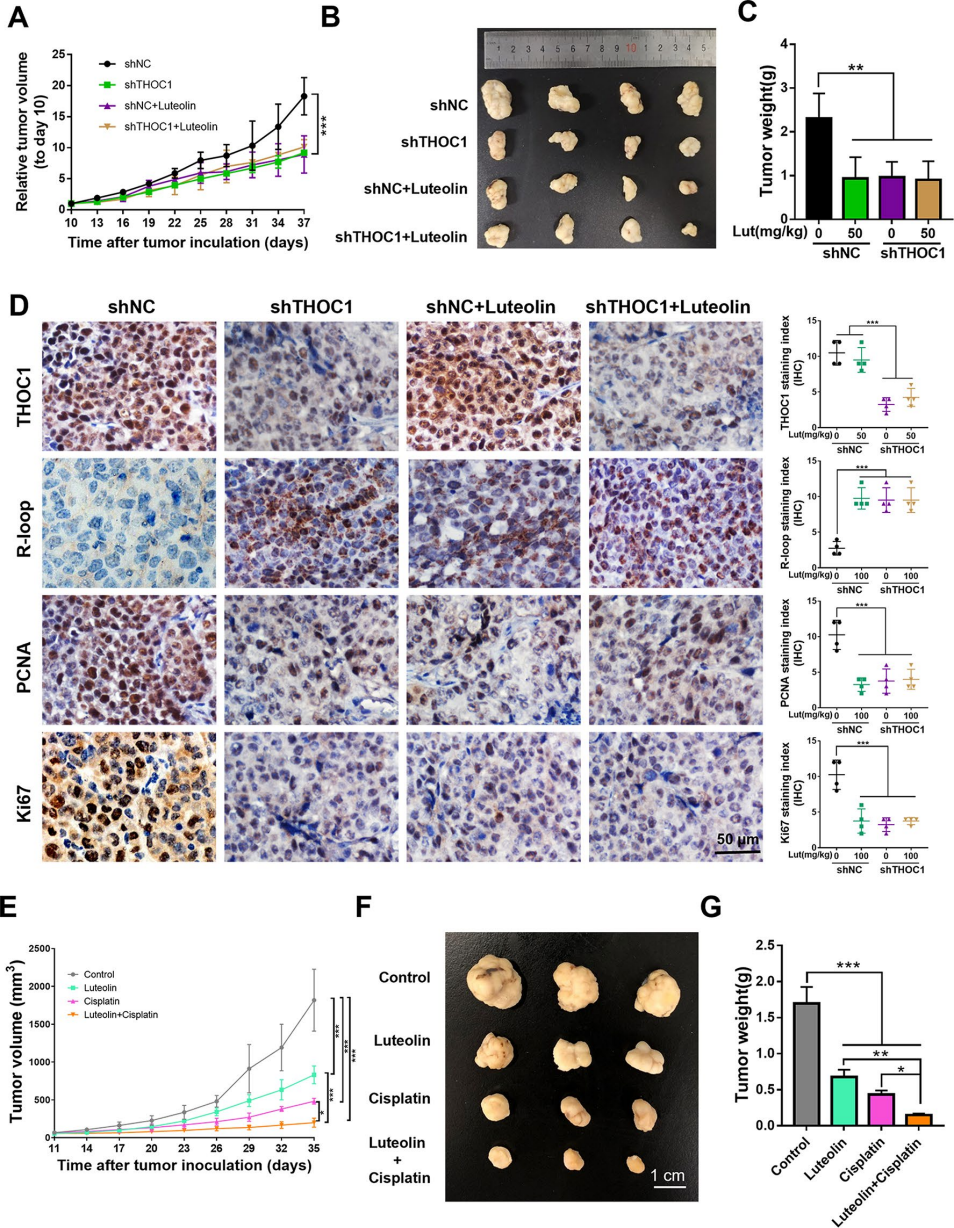
Luteolin reduces HCC proliferation by targeting THOC1 in vivo and enhances the anti-tumor effect of cisplatin. **a** Tumor growth curve, (**b**) representative images of tumor, and (**c**) tumor weight of PLC/PRF/5 cells stably transfected with shTHOC1 or shNC in BALB/c nu/nu mice treated with 50 mg/kg luteolin or saline as control, respectively (one-way ANOVA; **P < 0.01, ***P < 0.001). **d** immunohistochemistry staining indicates the expressions of THOC1, R-loop, and proliferation markers (PCNA and Ki67) in tumors (one-way ANOVA; ***P < 0.001). **e** Tumor growth curve, (**f**) representative images of tumor, and (**g**) tumor weight of PLC/PRF/5-bearing BALB/c nu/nu mice. Luteolin or cisplatin treatment significantly suppressed tumor growth. Furthermore, luteolin can enhance the antitumor effect of cisplatin (one-way ANOVA; *P < 0.05, **P < 0.01, ***P < 0.001)
