# Supplementary material for: Knockdown of THOC1 reduces the proliferation of hepatocellular carcinoma and increases the sensitivity to cisplatin
Source: J Exp Clin Cancer Res. 2020 Jul 15;39:135. doi: 10.1186/s13046-020-01634-7 (PMC7362638; doi:10.1186/s13046-020-01634-7)
Supplement: Supplementary file 1 — Additional file 1: Figure S1. Expression of THOC1 is positively correlated with the expression of proliferation markers (PCNA and Ki67). A. Representative IHC staining images for PCNA and Ki67 in THOC1-negative and -positive HCC tissues (scale bar = 50 μm). B. PCNA and THOC1 staining was quantified, and the correlation was analyzed (correlation coefficient: r = 0.6046, P < 0.01). C. Ki67 and THOC1 staining was quantified, and the correlation was analyzed (correlation coefficient: r = 0.5526, P < 0.01). Figure S2. Effects of cisplatin in HepG2/DDP-resistant cell lines after THOC1 knockdown. A. Western blot analysis was performed to analyze the expression levels of THOC1 in HepG2 and HepG2/DDP-resistant cell lines. B. Cell viability in the HepG2/DDP-resistant cell line after THOC1 knockdown was assessed via CCK-8 assays. **** P < 0.0001. [file 13046_2020_1634_MOESM1_ESM.docx]

**Expression of THOC1 is positively correlated with the expression of proliferation markers (PCNA and Ki67).**

We detected the expression of THOC1 and proliferation markers (PCNA and Ki67) in HCC tissue from 26 cases collected from Tianjin Medical General Hospital and Tumor Hospital of Tianjin within 5 years. Immunohistochemical staining showed that patients with HCC and high THOC1 expression levels also had high PCNA and Ki67 expression levels (Fig. S1A). Pearson’s correlation and linear regression analyses showed that the expression levels of THOC1 and proliferation markers (PCNA and Ki67) were positively correlated (Figs. S1 B and C).


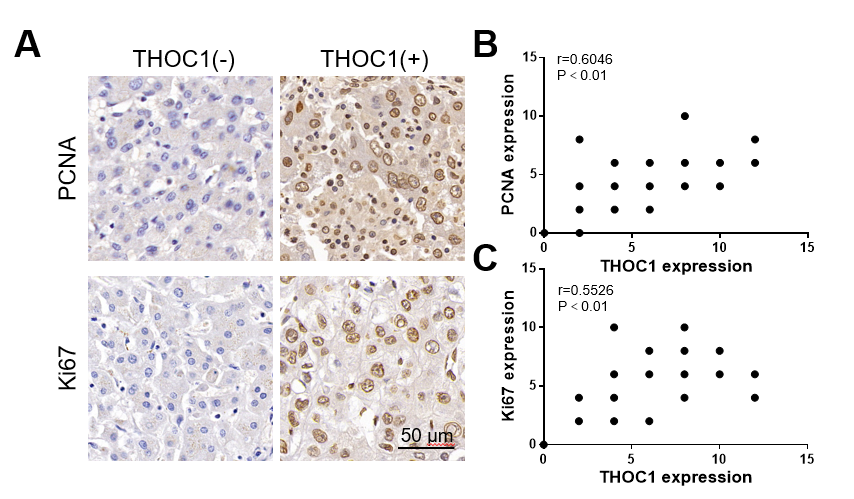


Figure S1. Expression of THOC1 is positively correlated with the expression of proliferation markers (PCNA and Ki67).

A. Representative IHC staining images for PCNA and Ki67 in THOC1-negative and -positive HCC tissues (scale bar = 50 μm). B. PCNA and THOC1 staining was quantified, and the correlation was analyzed (correlation coefficient: r = 0.6046, P < 0.01). C. Ki67 and THOC1 staining was quantified, and the correlation was analyzed (correlation coefficient: r = 0.5526, P < 0.01).

**Effects of cisplatin in HepG2/DDP-resistant cell lines after THOC1 knockdown.**

We detected the expression of THOC1 in HepG2/DDP-resistant cell lines. We found that the expression of THOC1 was higher in HepG2/DDP-resistant cell lines than in HepG2 (Fig. S2A). We further analyzed cell viability in HepG2/DDP-resistant cell lines after THOC1 knockdown. Interestingly, CCK-8 assays showed that the knockdown of THOC1 could strengthen DDP-induced cytotoxicity and impaired DDP resistance in HepG2 cell lines (Fig. S2B).


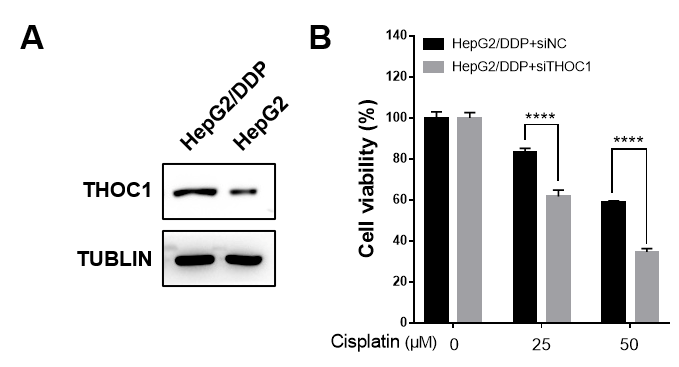


Figure S2. Effects of cisplatin in HepG2/DDP-resistant cell lines after THOC1 knockdown.

A. Western blot analysis was performed to analyze the expression levels of THOC1 in HepG2 and HepG2/DDP-resistant cell lines. B. Cell viability in the HepG2/DDP-resistant cell line after THOC1 knockdown was assessed via CCK-8 assays. **** *P* < 0.0001.
