# Supplementary material for: Knockdown of THOC1 reduces the proliferation of hepatocellular carcinoma and increases the sensitivity to cisplatin
Source: J Exp Clin Cancer Res. 2020 Jul 15;39:135. doi: 10.1186/s13046-020-01634-7 (PMC7362638; doi:10.1186/s13046-020-01634-7)
Supplement: Supplementary file 2 — Additional file 2: Table S1. Primers used for RT-PCR. [file 13046_2020_1634_MOESM2_ESM.pdf]

Supplemental Table 1: Primers used for RT-PCR

Gene	Oligonucleotide sequence (5’-3’)
THOC1	Forward: TCTTCTGTGGACGGATTCAGC
	Reverse: CTCGTCTCCCATTTCGCCTTC
GAPDH	Forward: GGAGCGAGATCCCTCCAAAAT
	Reverse: GGCTGTTGTCATACTTCTCATGG
