# Supplementary material for: Knockdown of THOC1 reduces the proliferation of hepatocellular carcinoma and increases the sensitivity to cisplatin
Source: J Exp Clin Cancer Res. 2020 Jul 15;39:135. doi: 10.1186/s13046-020-01634-7 (PMC7362638; doi:10.1186/s13046-020-01634-7)
Supplement: Supplementary file 3 — Additional file 3. [file 13046_2020_1634_MOESM3_ESM.pdf]

Report of Human Cell Line Authentication

（Notice:This authentication report is restricted to the cell sold from Guangzhou Cellcook Biotech Co., Ltd, and the date with seal is the date of delivery. ）

Ⅰ. Sample

Sample Name: labeled as 'Hep3B'

Ⅱ. Method and Procedure

1. PCR is amplified with STR Multi-amplification Kit (PowerPlex™16HS System);
2. PCR products are assayed with 3100 DNA Analyzer (Applied Biosystems®).
3. Amplification of gene COX1 and electrophoresis are employed to survey the species of the sample.

Ⅲ. Results

1. The STR profiles of the cell line sample are in the attached table and figure.
2. The search result in ATCC and DSMZ databases.
3. The electrophoresis figure of gene COX1.

Hep3B: ①No loci has tri-alleles or tetra-alleles. Contamination of other human cell line is not found (Figure 1 & Table 1). ②100% matched cell lines are found in ATCC and DSMZ data banks. And the cell line named as "Hep-3B" *et al.* (Figure 2 & Figure 3).③The sample is a human cell line. Contamination of other species cells are not found in the sample (Figure 4).

GUANGZHOU CELLCOOK BIOTECH CO.,LTD
25 FEB.2019
CELL STR PROFILING
PASS

Operator: Xiaohua Mo Auditor: Xuanyi Liang

Guangzhou Cellcook Biotech Co., Ltd

Figure 1. STR profiles of Hep3B cell line

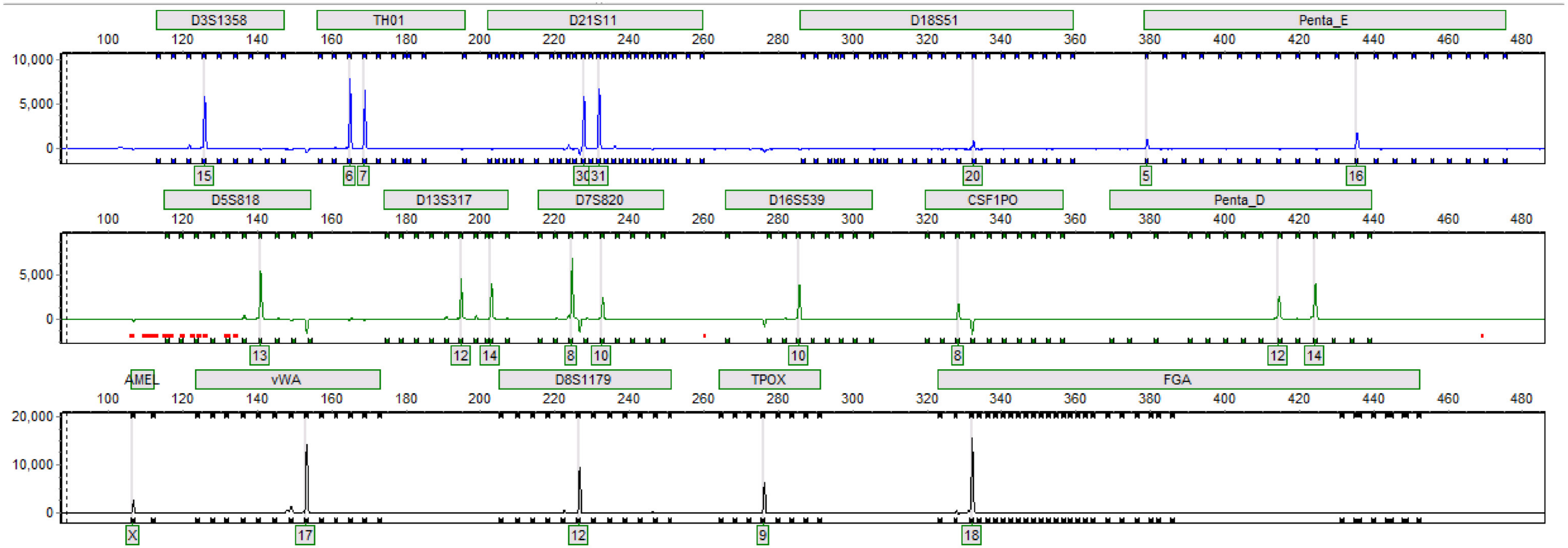

Table 1. STR profiles of Hep3B cell line

	Allele1	Allele2
D3S1358	15	
TH01	6	7
D21S11	30	31
D18S51	20	
Penta_E	5	16
D5S818	13	
D13S317	12	14
D7S820	8	10
D16S539	10	
CSF1PO	8	
Penta_D	12	14
AMEL	x	
vWA	17	
D8S1179	12	
TPOX	9	
FGA	18	

Figure 2. Search result in ATCC database

SEARCH THE STR DATABASE

As part of our continuing efforts to characterize and authenticate the cell lines in the Cell Biology collection, ATCC has developed a comprehensive database of short tandem repeat (STR) DNA profiles for all of our human cell lines. View our brief tutorial before starting.

1. STR Profiling Analysis
2. Matching Algorithm
3. Interrogating the Database

Showing 1 - 1 Of 1 PageSize: 100

Add to Cart	%Match	ATCC® Number	Designation	D5S818	D13S317	D7S820	D16S539	vWA	TH01	AMEL	TPOX	CSF1PO
☐	100	HB-8064	Hep 3B	13	12,14	8,10	10	17	6,7	X	9	8

Add to Cart Export to Excel

Disclaimer: Reference to this database and the data contained therein may be cited in publications, and ATCC encourages such citation or reference. While every reasonable effort has been made to assure the accuracy of these data, no warranty, express or implied, is made by ATCC as to their accuracy.

While ATCC has largely used the Promega PowerPlex® 1.2 System in the creation of these data and recommends that researchers wishing to produce data for comparison also use a Promega PowerPlex® System ATCC does not provide a general endorsement of this product or provide any warranty or representation regarding its quality or performance in the scientific community for the identification of human cell lines.

Figure 3. Search result in DSMZ database

Result of STR matching analysis by your data.

- DSMZ Profile Database -

A graphical presentation is shown at the bottom of this page.

EV	Cell No.	Cell name	Locus names									Figures
			D5S818	D13S317	D7S820	D16S539	VWA	TH01	AM	TPOX	CSF1PO	
	Query (Your Cell)		*13*	*12,14*	*8,10*	*10*	*17*	*6,7*	*x*	*9*	*8*	
1.20(36/30)	93	HEP-3B	13,13	12,14	8,10	10,10	17,17	6,7	X,X	9,9	8,8	-
1.20(36/30)	HB-8064	Hep 3B2.1-7 [Hep 3B, Hep-3B, Hep3B]	13,13	12,14	8,10	10,10	17,17	6,7	X,X	9,9	8,8	-
0.80(24/30)	CRL-5966	NCI-BL2107	13,13	12,13	8,8	10,13	17,17	6,7	X,Y	10,11	8,11	-
0.73(22/30)	490	MUTZ-5	11,11	10,12	10,10	10,10	16,17	7,7	X,X	8,11	8,9	-
0.73(22/30)	CCL-205	CCD 18Lu	12,12	12,12	8,8	12,13	15,17	6,7	X,X	8,11	8,8	-
0.73(22/30)	CRL-1459	CCD-18Co	12,12	12,12	8,8	12,13	15,17	6,7	X,X	8,11	8,8	-
0.73(22/30)	CRL-2320	HCC1008	13,13	12,12	10,10	14,15	17,19	7,7	X,X	11,11	12,13	-
[illegible]	[illegible]	[illegible]	[illegible]	[illegible]	[illegible]	[illegible]	[illegible]	[illegible]	[illegible]	[illegible]	[illegible]	[illegible]

Figure 4. Authentication of the species of the sample

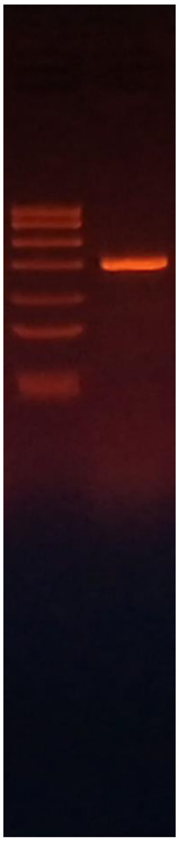

M: Marker. As the size of 700, 600, 500, 400, 300, 200 and 100bp from up to down.

Nine species are checked, as follow: *Homo sapiens* 391bp, *Cricetulus griseus*315bp, *Macaca mulatta*287bp, *Cercopithecus aethiops*222bp, *Rattus norvegicus*196bp, *Canis familiaris*172bp, *Mus musculus*150bp, *Bos Taurus*102bp, IC 70bp

The sample: The band size is 391bp which matches the size of human.

Applied Biosystems
GeneMapper® ID-X 1.4

Project: 201405304

Sample Name	Marker	OS	BIN	PHR	LPH	MPH	SPK	AN	BD	CC	GQ
HepG2-A	D3S1358									NA	
HepG2-A	TH01			NA						NA	
HepG2-A	D21S11									NA	
HepG2-A	D18S51									NA	
HepG2-A	Penta E									NA	
HepG2-A	D5S818									NA	
HepG2-A	D13S317									NA	
HepG2-A	D7S820			NA						NA	
HepG2-A	D16S539									NA	

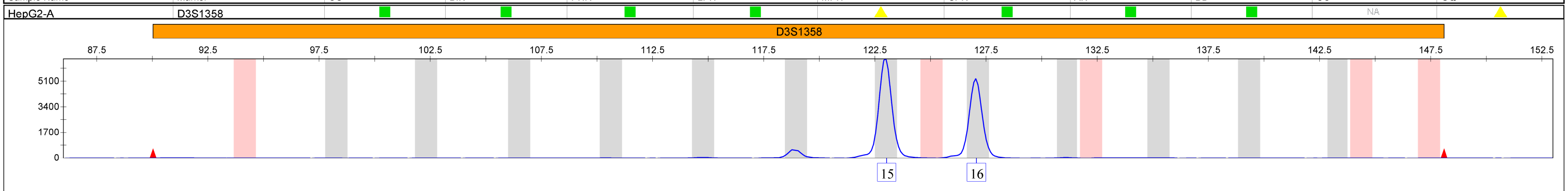

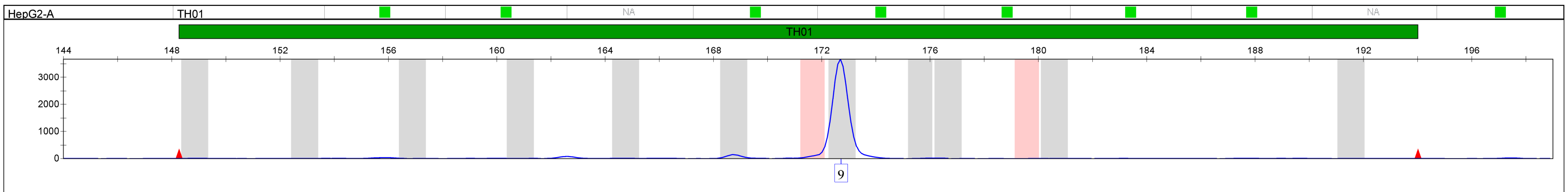

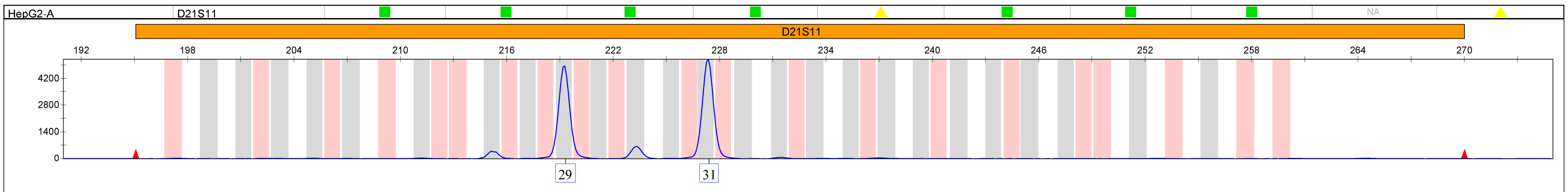

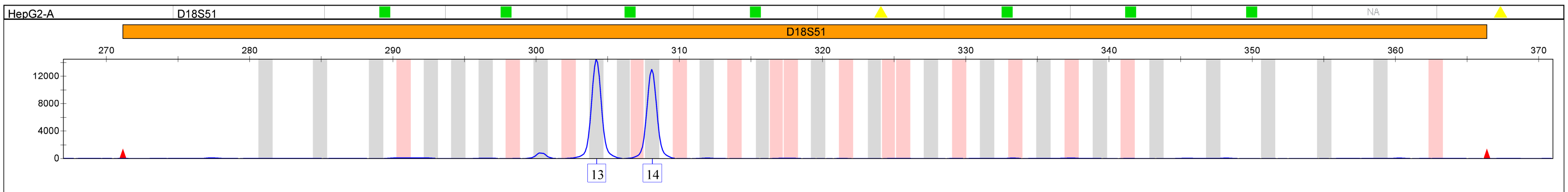

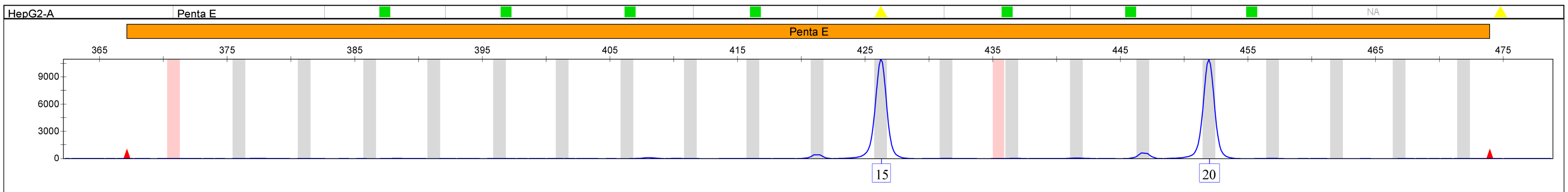

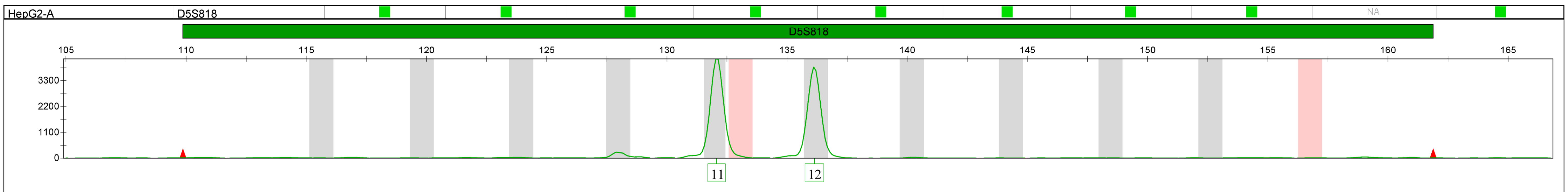

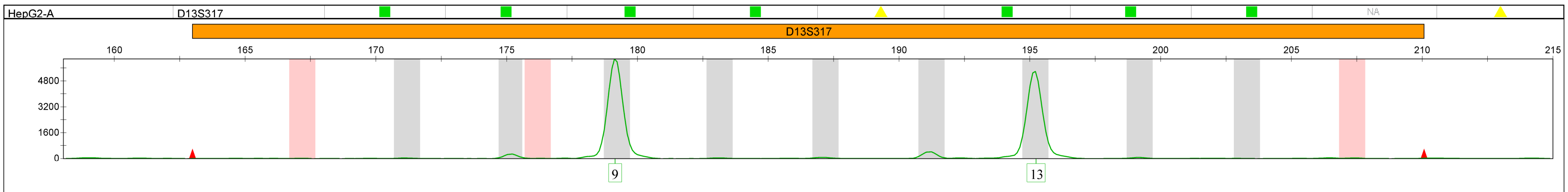

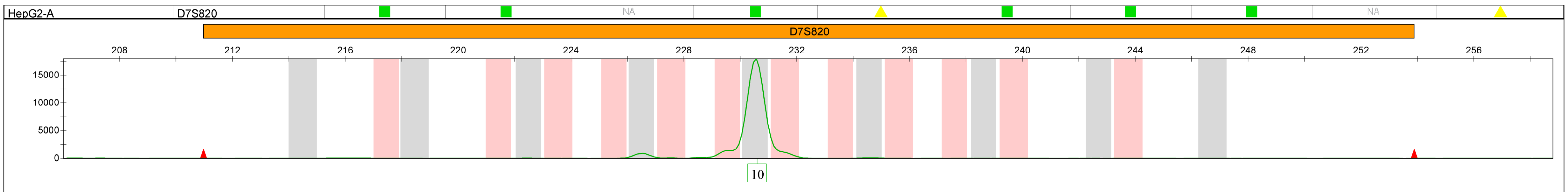

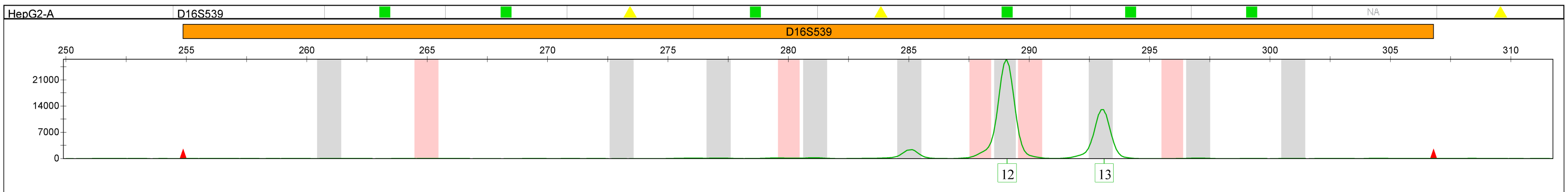

Sample Name	Marker	OS	BIN	PHR	LPH	MPH	SPK	AN	BD	CC	GQ
HepG2-A	CSF1PO									NA	

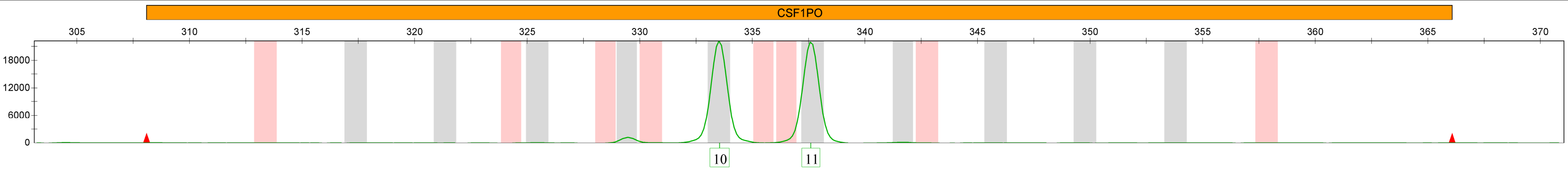

HepG2-A	Penta D									NA	

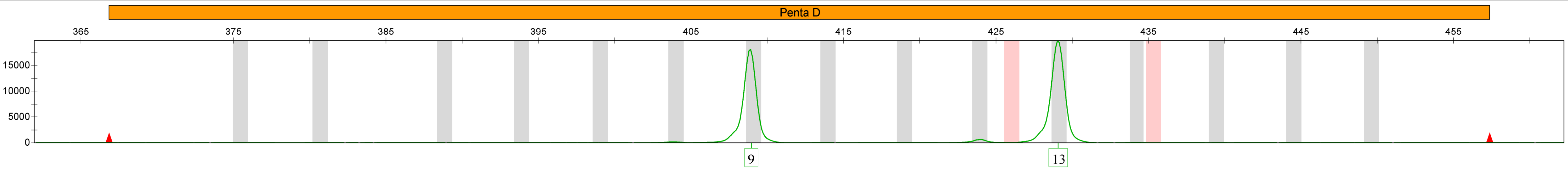

HepG2-A	AMEL									NA	

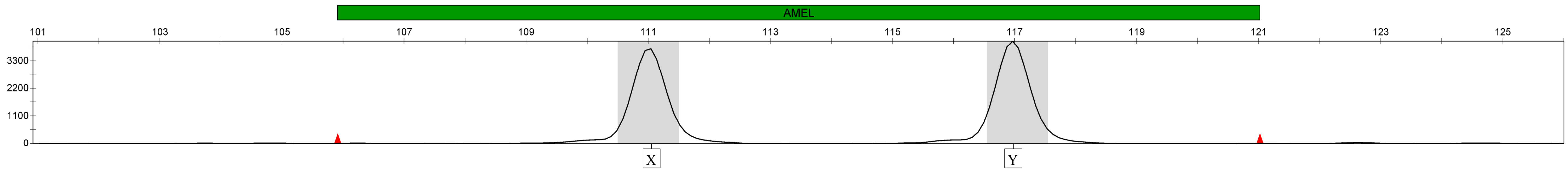

HepG2-A	vWA			NA						NA	

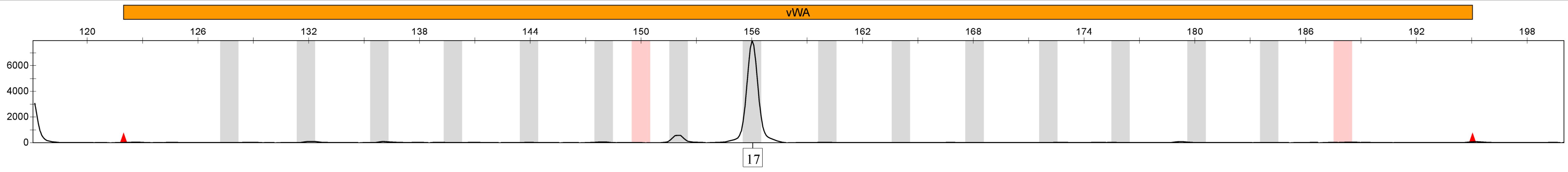

HepG2-A	D8S1179									NA	

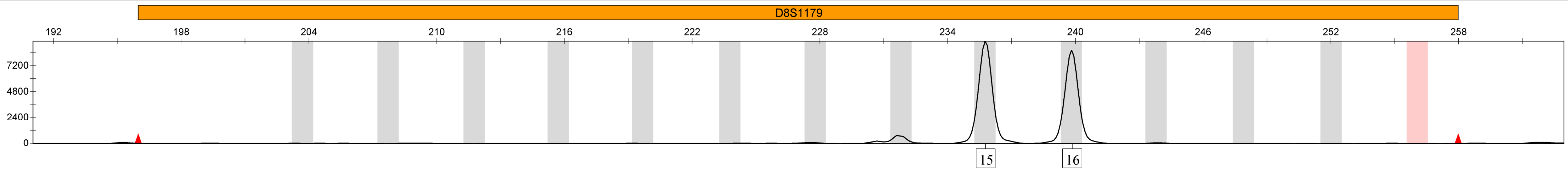

HepG2-A	TPOX									NA	

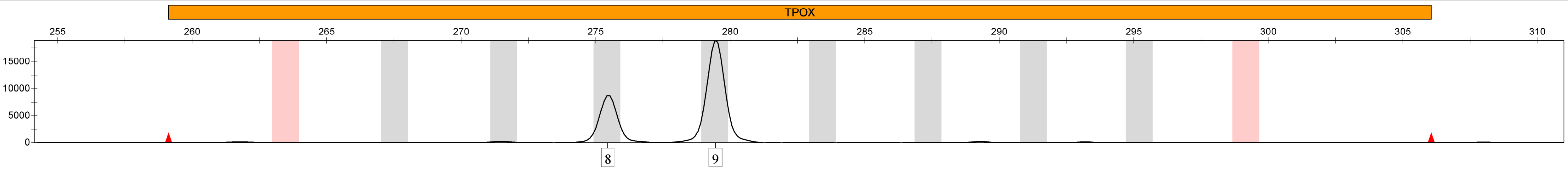

HepG2-A	FGA									NA	

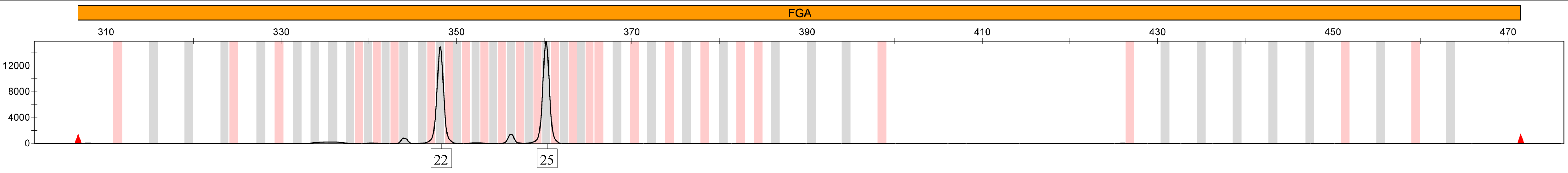

HepG2-A	D19S433			NA						NA	

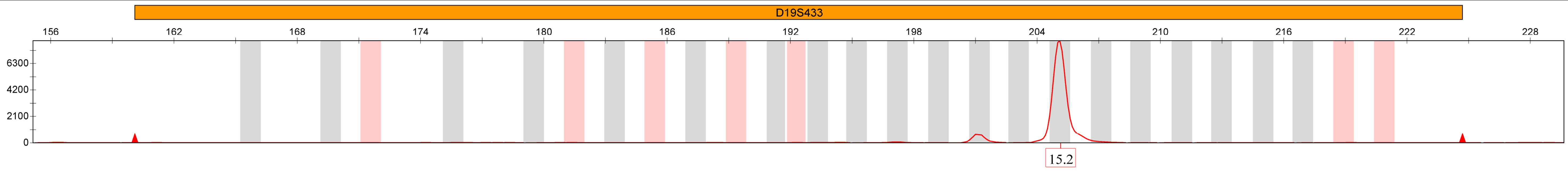

HepG2-A	D2S1338									NA	

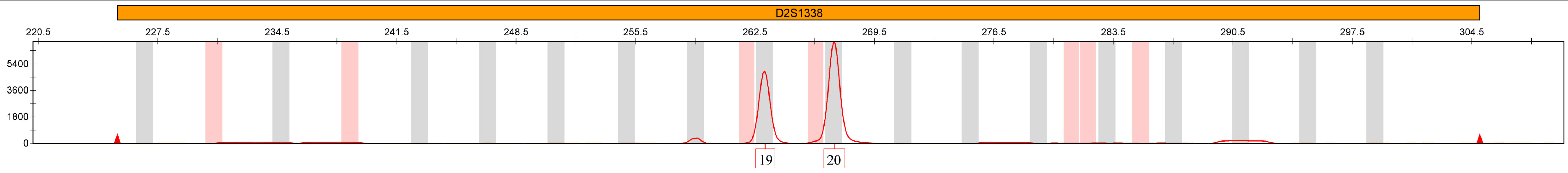

Sample File	Sample Name	Panel	SQI	OS	SQ
68_D09_CellLineAuthentication-1-0626.fsa	plclfrt15	STR Profile 1-human			

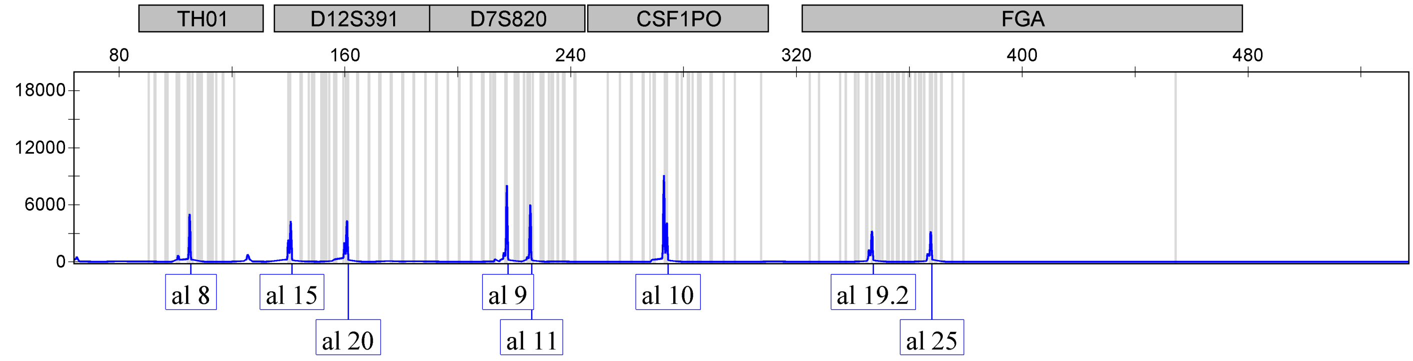

Sample File	Sample Name	Panel	SQI	OS	SQ
82_B11_CellLineAuthentication-0627.fsa	plclfrt15	STR Profile 2-human			

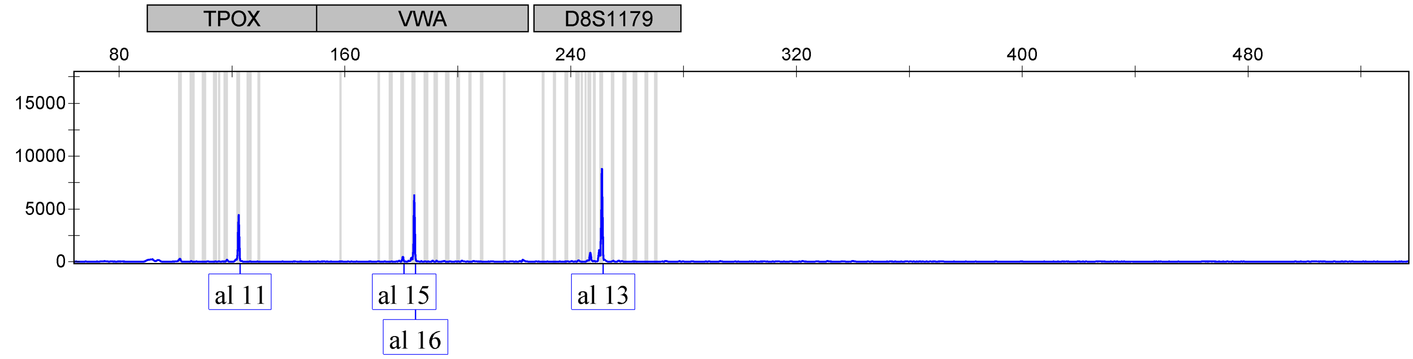

Sample File	Sample Name	Panel	SQI	OS	SQ
68_D09_CellLineAuthentication-4-0626.FSA	plclfrt15	STR Profile 3-human			

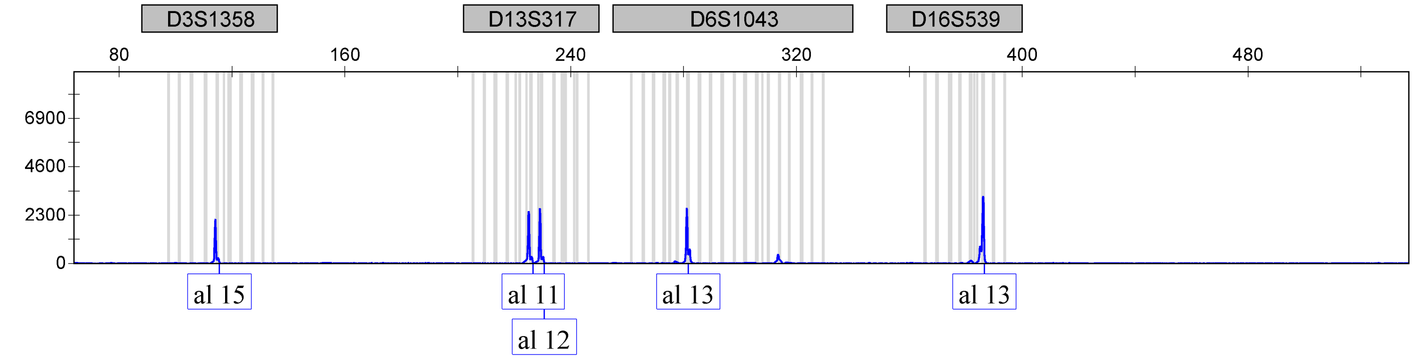

Sample File	Sample Name	Panel	SQI	OS	SQ
92_D12_CellLineAuthentication-4-0626.FSA	plclfrt15	STR Profile 4-human			

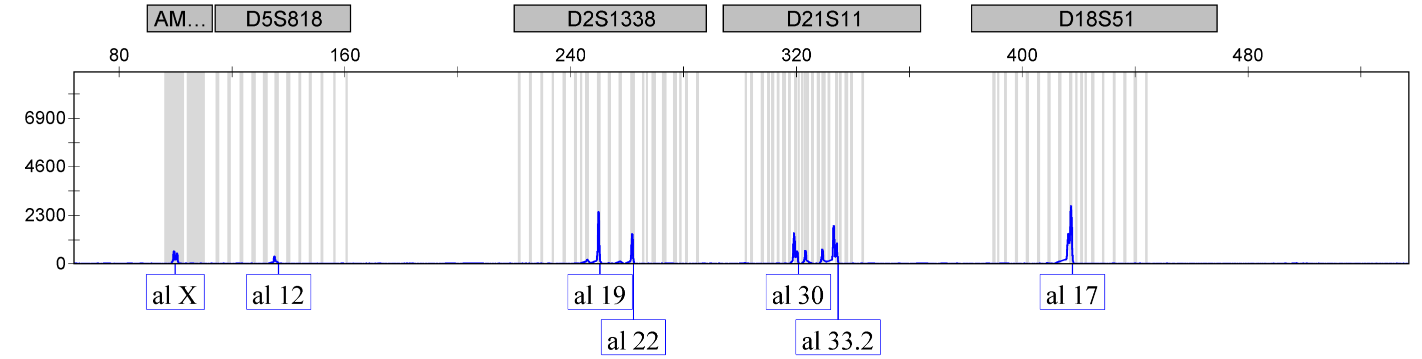

0622cell

GeneMapper 4.0

Sample File	Sample Name	Panel	SQI	OS	SQ
68_D09_CellLineAuthentication-1-0626.fsa	plclfrt15	STR Profile 1-human			

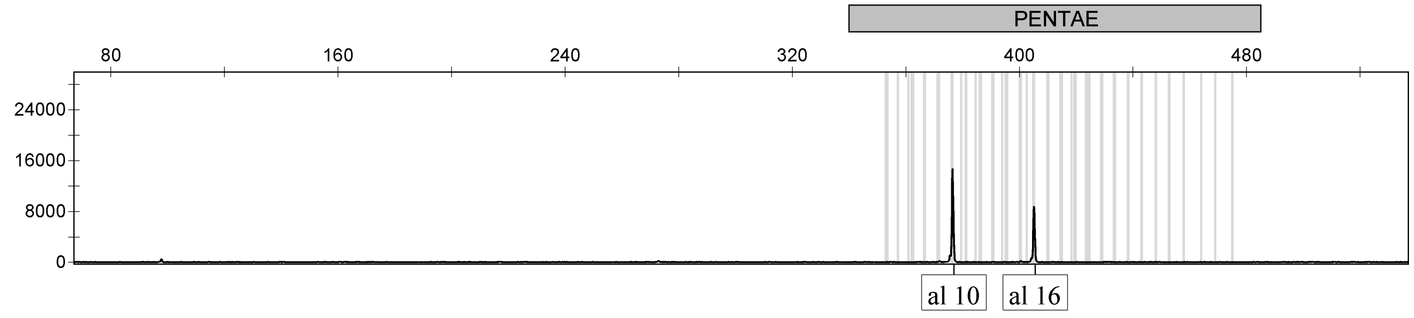

68_D09_CellLineAuthentication-3-0626.fsa	plclfrt15	STR Profile 2-human			

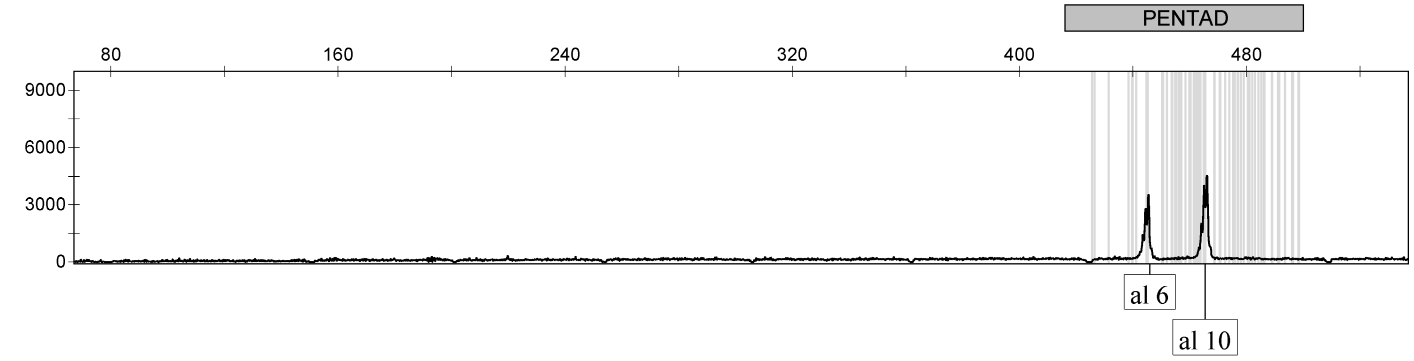

68_D09_CellLineAuthentication-4-0626.FSA	plclfrt15	STR Profile 3-human			

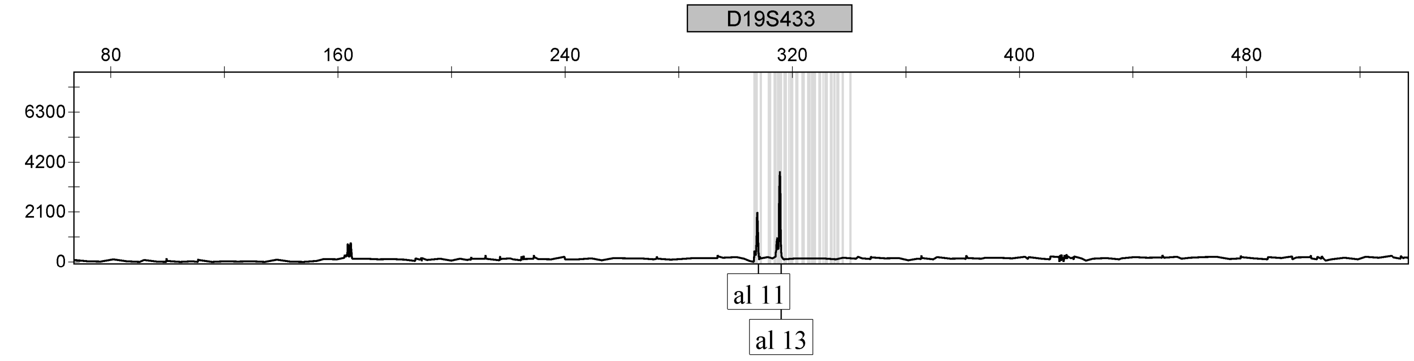

92_D12_CellLineAuthentication-4-0626.FSA	plclfrt15	STR Profile 4-human			

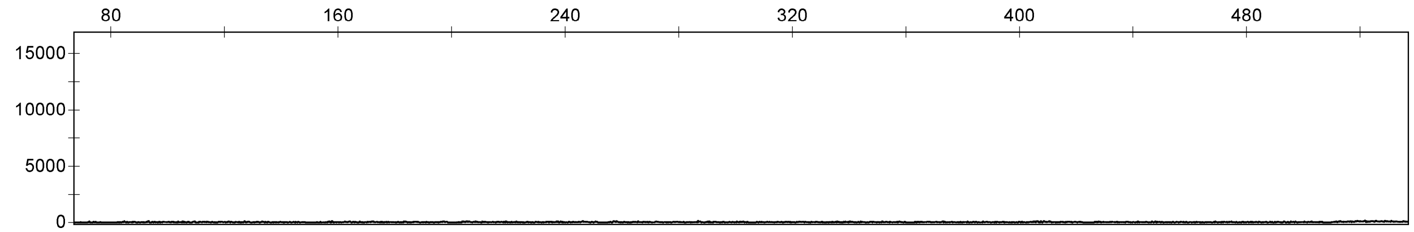

Report of Human Cell Line Authentication

（Notice:This authentication report is restricted to the cell sold from Guangzhou Cellcook Biotech Co., Ltd, and the date with seal is the date of delivery. ）

Ⅰ. Sample

Sample Name: labeled as ‘SK-HEP-1’

Ⅱ. Method and Procedure

1. PCR is amplified with STR Multi-amplification Kit (PowerPlex™16HS System);
2. PCR products are assayed with 3100 DNA Analyzer (Applied Biosystems®).
3. Amplification of gene COX1 and electrophoresis are employed to survey the species of the sample.

Ⅲ. Results

1. The STR profiles of the cell line sample are in the attached table and figure.
2. The search result in ATCC and DSMZ databases.
3. The electrophoresis figure of gene COX1.

SK-HEP-1: ①No loci has tri-alleles or tetra-alleles. Contamination of other human cell line is not found (Figure 1 & Table 1). ②100% matched cell lines are found in ATCC and DSMZ data banks. And the cell line named as “SK-HEP-1:” *et al.* (Figure 2 & Figure 3).③The sample is a human cell line. Contamination of other species cells are not found in the sample (Figure 4).

GUANGZHOU CELLCOOK BIOTECH CO.,LTD
25 FEB 2019
CELL STR PROFILING
PASS

Operator: Xiaohua Mo Auditor: Xuanyi Liang

Guangzhou Cellcook Biotech Co., Ltd

Figure 1. STR profiles of SK-HEP-1cell line

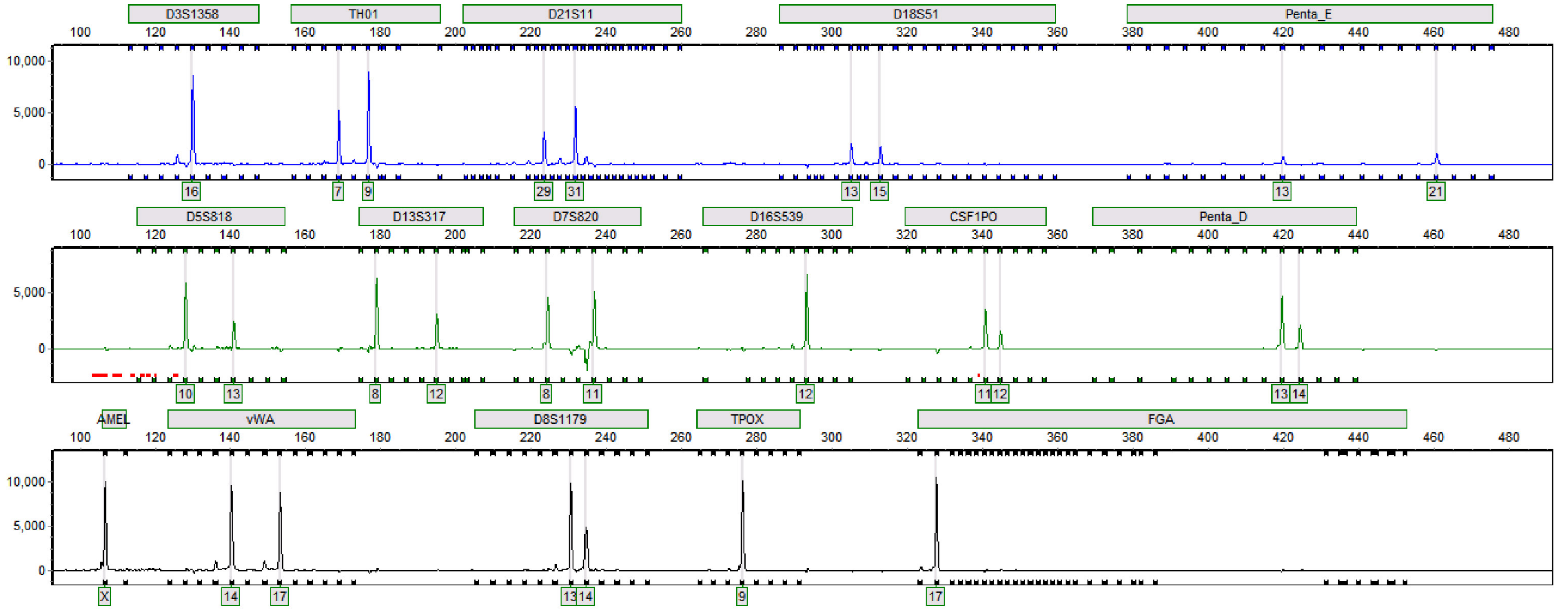

Table 1. STR profiles of SK-HEP-1 cell line

	Allele1	Allele2
D3S1358	16	
TH01	7	9
D21S11	29	31
D18S51	13	15
Penta_E	13	21
D5S818	10	13
D13S317	8	12
D7S820	8	11
D16S539	12	
CSF1PO	11	12
Penta_D	13	14
AMEL	x	
vWA	14	17
D8S1179	13	14
TPOX	9	
FGA	17	

Figure 2. Search result in ATCC database

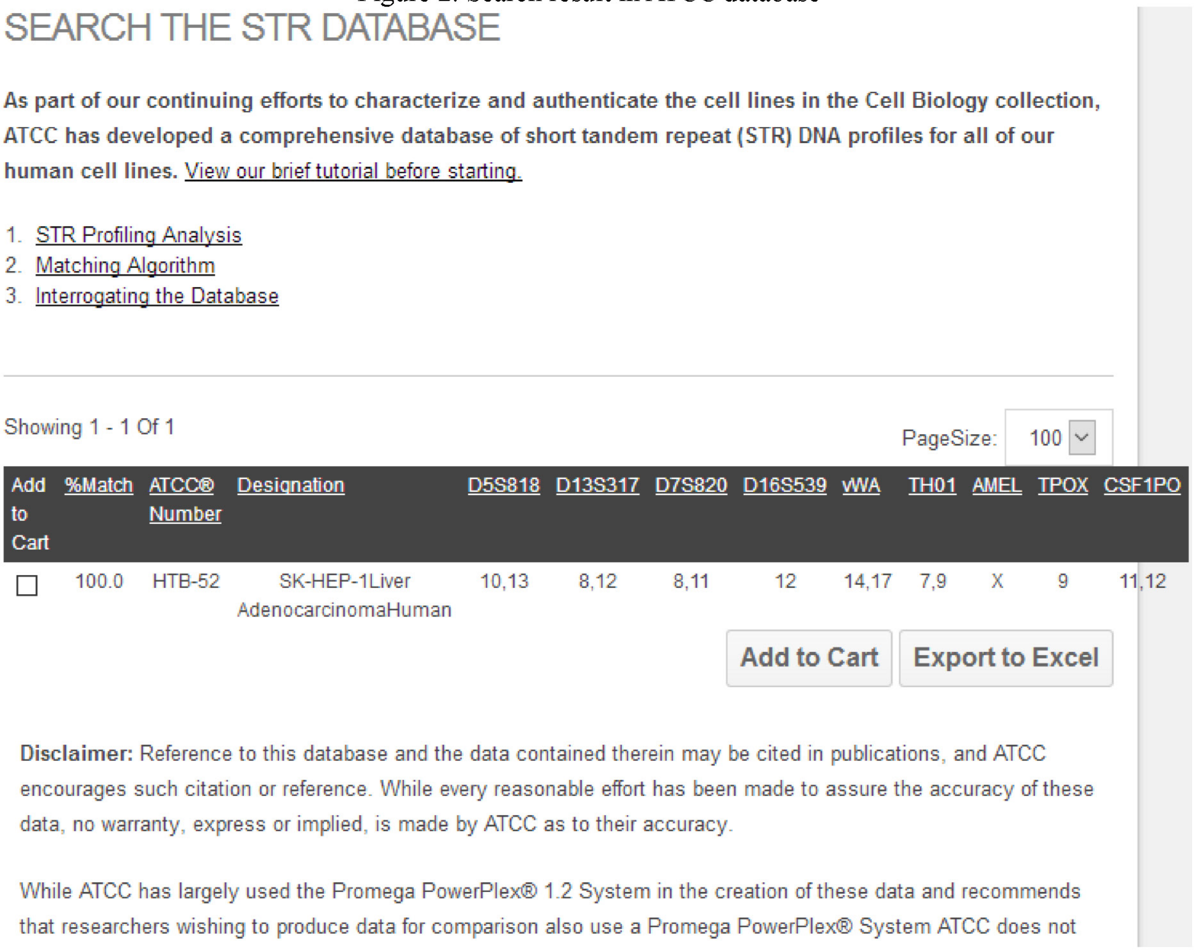

SEARCH THE STR DATABASE

As part of our continuing efforts to characterize and authenticate the cell lines in the Cell Biology collection, ATCC has developed a comprehensive database of short tandem repeat (STR) DNA profiles for all of our human cell lines. View our brief tutorial before starting.

1. STR Profiling Analysis
2. Matching Algorithm
3. Interrogating the Database

Showing 1 - 1 Of 1

PageSize: 100

Add to Cart	%Match	ATCC® Number	Designation	D5S818	D13S317	D7S820	D16S539	vWA	TH01	AMEL	TPOX	CSF1PO
☐	100.0	HTB-52	SK-HEP-1Liver AdenocarcinomaHuman	10,13	8,12	8,11	12	14,17	7,9	X	9	11,12

Add to Cart Export to Excel

Disclaimer: Reference to this database and the data contained therein may be cited in publications, and ATCC encourages such citation or reference. While every reasonable effort has been made to assure the accuracy of these data, no warranty, express or implied, is made by ATCC as to their accuracy.

While ATCC has largely used the Promega PowerPlex® 1.2 System in the creation of these data and recommends that researchers wishing to produce data for comparison also use a Promega PowerPlex® System ATCC does not

Figure 3. Search result in DSMZ database

Result of STR matching analysis by your data.

- DSMZ Profile Database -

A graphical presentation is shown at the bottom of this page.

EV	Cell No.	Cell name	Locus names									Figures
			D5S818	D13S317	D7S820	D16S539	VWA	TH01	AM	TPOX	CSF1PO	
	Query (Your Cell)		*10,13*	*8,12*	*8,11*	*12*	*14,17*	*7,9*	*X*	*9*	*11,12*	
1.09(36/33)	141	SK-HEP-1	10,13	8,12	8,11	12,12	14,17	7,9	X,X	9,9	11,12	-
1.09(36/33)	HTB-52	SK-HEP-1	10,13	8,12	8,11	12,12	14,17	7,9	X,X	9,9	11,12	-
0.91(30/33)	JCRB1131	UE6E7T-1	10,13	8,8	8,11	11,12	17,18	9,9	X,X	9,11	11,12	-
0.91(30/33)	JCRB1133	UE6E7T-2	10,13	8,8	8,11	11,12	17,18	9,9	X,X	9,11	11,12	-
0.91(30/33)	JCRB1136	UE6E7T-3	10,13	8,8	8,11	11,12	17,18	9,9	X,X	9,11	11,12	-
0.91(30/33)	JCRB1140	UBE6T-6	10,13	8,8	8,11	11,12	17,18	9,9	X,X	9,11	11,12	-
0.91(30/33)	JCRB1147	UE7T-9	10,13	8,8	8,11	11,12	17,18	9,9	X,X	9,11	11,12	-
0.91(30/33)	JCRB1149	UE6E7T-11	10,13	8,8	8,11	11,12	17,18	9,9	X,X	9,11	11,12	-
0.91(30/33)	JCRB1151	UE6E7T-12	10,13	8,8	8,11	11,12	17,18	9,9	X,X	9,11	11,12	-
0.91(30/33)	JCRB1154	UE7T-13	10,13	8,8	8,11	11,12	17,18	9,9	X,X	9,11	11,12	-
0.91(30/33)	JCRB1159	UBE6T-15	10,13	8,8	8,11	11,12	17,18	9,9	X,X	9,11	11,12	-
[illegible]	[illegible]	[illegible]	[illegible]	[illegible]	[illegible]	[illegible]	[illegible]	[illegible]	[illegible]	[illegible]	[illegible]	[illegible]

Figure 4. Authentication of the species of the sample

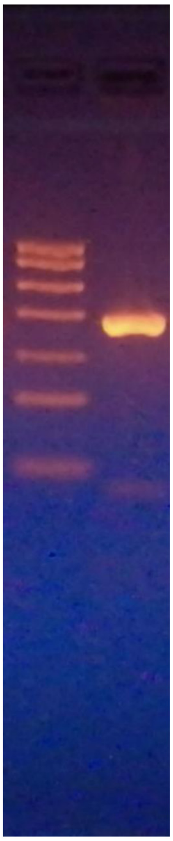

M: Marker. As the size of 700, 600, 500, 400, 300, 200 and 100bp from up to down.

Nine species are checked, as follow: *Homo sapiens* 391bp, *Cricetulus griseus*315bp, *Macaca mulatta*287bp, *Cercopithecus aethiops*222bp, *Rattus norvegicus*196bp, *Canis familiaris*172bp, *Mus musculus*150bp, *Bos Taurus*102bp, IC 70bp

The sample: The band size is 391bp which matches the size of human.
